# The circular RNA circ-GRB10 participates in the molecular circuitry inhibiting human intervertebral disc degeneration

**DOI:** 10.1038/s41419-020-02882-3

**Published:** 2020-08-13

**Authors:** Wei Guo, Kun Mu, Bin Zhang, Chao Sun, Ling Zhao, Hao-Ran Li, Zhan-Yin Dong, Qing Cui

**Affiliations:** 1 Department of Orthopaedics, Hebei Province Cangzhou Hospital of Integrated Traditional and Western Medicine (Cangzhou No.2 Hospital), 31 Huanghe Road, 061001 Cangzhou, Hebei Province P. R. China; 2 Department of Breast Surgery, Hebei Province Cangzhou Hospital of Integrated Traditional and Western Medicine (Cangzhou No.2 Hospital), 31 Huanghe Road, 061001 Cangzhou, Hebei Province P. R. China; 3 Department of Orthopaedics, Tianjin Medical University General Hospital, 154 Anshan Road, Heping District, 300052 Tianjin, P. R. China

**Keywords:** Mechanisms of disease, Diseases

## Abstract

Intervertebral disc degeneration (IDD) is the most common degenerative disease all over the word. Our previous study confirmed that the downregulated circ-GRB10 directly interacts with miR-328-5p, which modulate ERBB2 and leads to the degeneration of intervertebral disc; however, the underpinning mechanism of circ-GRB10 dysregulation remains unclear. We identified that FUS and demonstrated that circ-GBR10 biosynthesis in nucleus pulposus (NP) cells was promoted by FUS, whose expression was controlled by miR-141-3p. In addition, ERBB2 downregulation led to decreased Erk1/2 phosphorylation which enhanced miR-141-3p production in NP cells. In vivo data indicated that circ-GRB10 inhibited IDD in rat model. The present study revealed that miR-141-3p and FUS are key factors that regulate circ-GRB10 synthesis in NP cells. In addition, circ-GBR10 participates in the molecular circuitry that controls human IDD development. These findings provide a basis for further functional, diagnostic and therapeutic studies of circ-GRB10 in IDD.

## Introduction

The Global Burden of Disease Study stated that low back pain (LBP) represents an important cause of disability worldwide^1^. LBP is tightly associated with intervertebral disc degeneration (IDD), which involves ~40% of all LBP cases, causing significant economic and social burdens worldwide^2,3^. According to previous reports, 84% of the world population have low back pain during their lifetime, with 10% being chronically disabled^4^.

Currently, IDD pathogenesis is largely unclear; however, it could be due to microenvironmental alterations in the intervertebral discs caused by various factors such as genetic features, aging, sex, a predisposing injury, and the environment^5,6^. The main feature of IDD pathology is elevated biosynthesis of catabolic enzymes combined with reduced extracellular matrix (ECM) accumulation caused by imbalanced anabolism and catabolism^7^. Intervertebral discs comprise a central nucleus pulposus (NP), a peripheral annulus fibrosus (AF), and cartilaginous end plates, which connect overlying capillary beds cranially and caudally. The NP maintains homeostasis by producing an ECM mostly comprising type II collagen and proteoglycans, the main functional components of intervertebral discs, which are indispensable to maintain the disc height and absorb various mechanical loads^8^. It is well known that loss of collagen-II and aggrecan is an early critical event in the degenerative cascade in Intervertebral disc tissue^9^. MMP-13 is the most important enzymes that hydrolyze collagens^10^. ADAMTS-5 is classified as the major aggrecanases due to their high efficiency in cleaving aggrecan^11^. A large body of evidence supporting the involvement of MMP-13 and ADAMTS-5 in IDD pathogenesis^12^. During IDD, the main histological alteration involves the centrally located NP cells, which after a phenotypic transformation are substituted by smaller fibrochondrocyte-like cells, with reduced proteoglycan production and a global shift towards synthesizing fibrotic materials and compromising the structural integrity of discs^13,14^. Therefore, unveiling the mechanisms underpinning such imbalance is urgently needed for the development of new therapeutic targets in IDD.

Mounting evidence supports roles for circular RNAs (circRNAs) in IDD^15–17^. Previous research demonstrated that circRNAs are closed RNAs produced by back-splicing of single pre-mRNAs^18^. It is not completely known how circRNAs are biosynthesized, although complementarity between inverted sequences in flanking introns and the activity of RNA-binding proteins (RBPs) increase the contiguity of splice sites contributing to back-splicing in mammalian cells^19–22^.

The RBP FUS affects splicing regulation^23^ with many splicing factors termed FUS interactors^24–26^. FUS mutations could lead to protein mislocalization to the cytosol, with decreased nuclear FUS amounts and occurrence of abnormal cytosolic aggregates^27,28^. The FUS protein is involved in regulating intracellular RNA transport, mRNA synthesis, alternative splicing, and polyadenylation site selection^29^. He et al. demonstrated that FUS combined with circ_002136 and promoted the generation of circ_002136 in Glioma^30^. It was recently shown that FUS controls the expression of 19 circRNAs by binding to introns that flank the splicing junction^31^. Moreover, FUS was reported to be regulated by many miRNAs, including miR-141-3p^32,33^. Studies revealed miR-141-3p is upregulated in NP tissue specimens from IDD cases and demonstrated that miR-141-3p is associated with disc degeneration^34^. However, the function and mechanism of FUS, as well as the interaction between FUS and miR-141-3p in IDD have not been reported.

Our previous research confirmed that circ-GRB10 amounts are markedly reduced in NP cells from IDD patients, which accelerates IDD development by enhancing miR-328-5p mediated ERBB2 suppression in NP cells^15^. However, the mechanism of circ-GRB10 downregulation in degenerative NP cells remains unclear. In this study, we demonstrated that the miR-141-3p, which is significantly increased in degenerative NP cells^34^, regulate expression of the FUS, which is responsible for the generation of circ-GRB10 in NP cells. Furthermore, we showed that ERBB2 downregulation led to decreased Erk1/2 phosphorylation, and the decreased levels of Erk1/2 phosphorylation enhanced miR-141-3p biogenesis in degenerative NP cells, promoting IDD development. Taken together, these findings suggested circ-GBR10 contributes to the molecular circuitry controlling IDD development in humans.

## Results

### Circ-GRB10 regulates NP cell functions through the ERBB2/Erk signaling pathway

**Fig. 1:**
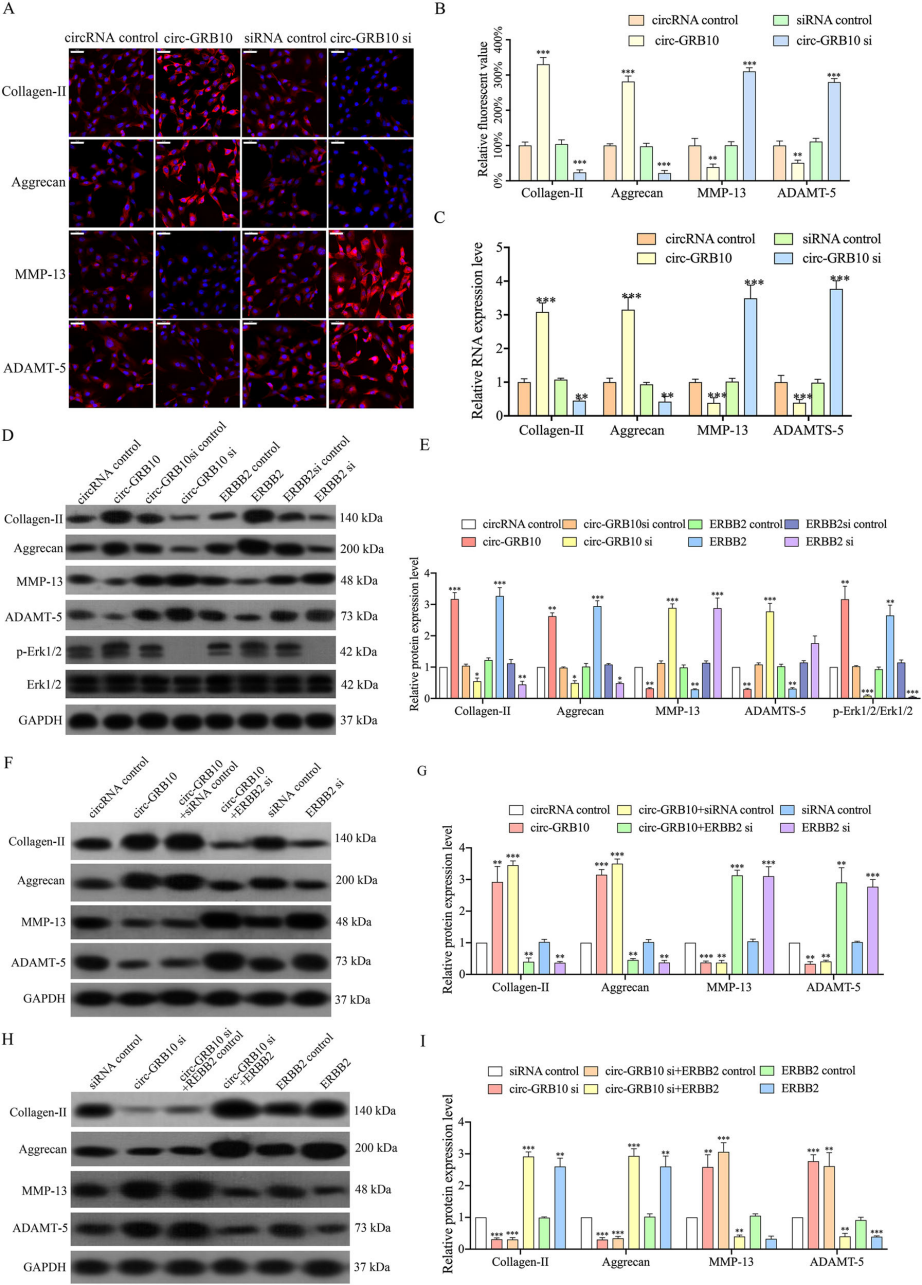
circ-GRB10 regulates NP cell functions through ERBB2/Erk signal pathway. **a** Collagen-II, aggrecan, MMP-13, ADAMT-5 expression were analyzed in circ-GRB10 or circ-GRB10 siRNA transfected cultured primary human NP cells using Immunofluorescence staining analysis. **b** The corresponding bar graphs show quantitative analysis of the relative fluorescent value of each group. n = 3 replicates per group, **p < 0.01, ***p < 0.001. Scale bar = 50 μm. **c** **q**RT-PCR showing the expression levels of collagen II, aggrecan, MMP13, ADAMT -5 in human NP cells after circ-GRB10 overexpression or knockdown. Three independent experiments are presented as mean ± SEM (error bars). **P < 0.01, ***P < 0.001. **d** The expression levels of Collagen-II, aggrecan, MMP13, ADAMT5, and p-Erk1/2 were detected by western blot; Quantitative analysis was shown in **e**, and three independent repeats were performed in each experiment. ***p < 0.001. **f** NP cell were co-transfected with circ-GRB10 and ERBB2 siRNA. Western blot assay showed that ERBB2 siRNA blocked the effect of circ-GRB10 on Collagen-II, aggrecan, MMP13 and ADAMT5 expression. Quantitative analysis was shown in **g**, and three independent repeats were performed in each experiment. ***p < 0.001. **h** NP cell were co-transfected with circ-GRB10 siRNA and ERBB2. Western blot assay showed that ERBB2 attenuated the effect of circ-GRB10 siRNA on Collagen-II, aggrecan, MMP13 and ADAMT5 expression. Quantitative analysis was shown in **i**, and three independent repeats were performed in each experiment. ***p < 0.001.

Our previous study revealed circ-GRB10 promotes NP cell survival by increasing ERBB2 amounts via suppression of miR-328-5p. However, the effect of circ-GRB10 expression on NP cell anabolism or catabolism remains obscure. To further assess circ-GBR10’s functions in IDD pathogenesis, circ-GRB10 or circ-GRB10 small interfering RNA (siRNA) was transiently transfected into cultured primary human NP cells. As shown in Supplementary Fig. S1, overexpression and knockdown of circ-GRB10 have no effect on linear GRB10, but only affect circular GRB10. The immunofluorescence results demonstrated that after overexpressing circ-GRB10 in NP cells, significantly upregulation of collagen II and aggrecan, and decreased amounts of MMP-13 and ADAMTS-5 were found. Conversely, circ-GRB10 knockdown resulted in opposite effects (Fig. 1a, b). These findings were confirmed by qRT-PCR (Fig. 1c).

Our previous research demonstrated circ-GRB10 inhibits IDD development by regulating ERBB2 expression in NP cells. Increasing evidence supports an important role for the ERBB2 gene and Erk signaling pathways in the progression of many human diseases^35–37^. Meanwhile, the Erk pathway is altered during IDD^38^, and plays a significant role in extracellular metabolism^39^. These results prompted us to assess the plausible association of circ-GRB10 with ERBB2/ Erk signaling. In this study, primary human NP cells underwent transfection with circ-GBR10, circ-GRB10 siRNA, and respective negative controls, respectively. As shown in Fig. 1d, e, western blot assay showed that p-Erk1/2, collagen II, and aggrecan amounts were significantly increased, while MMP-13 and AMADT-5 levels were reduced in NP cells overexpressing circ-GRB10. Conversely, p-Erk1/2, collagen II and aggrecan were downregulated, and MMP-13 and AMADT-5 amounts were increased in NP cells transfected with circ-GRB10 siRNA (Fig. 1d, e). Furthermore, ERBB2 affected p-Erk1/2, in a similar way as circ-GRB10 (Fig. 1d, e), suggesting cric-GRB10 modulates IDD progression via ERBB2/Erk signaling. Therefore, in order to further validated whether ERBB2 was the downstream mediator of circ-GRB10 in the NP cells. We cotransfected circ-GRB10 and ERBB2 siRNA into NP cells, and observed that the positive effects of circ-GRB10 on NP cells functions were attenuated in the absence of ERBB2 (Fig. 1f, g). Moreover, upregulation of ERBB2 counteracted the inhibitory effect of circ-GRB10 knockdown on NP cells function (Fig. 1h, i). Collectively, the above findings indicated that circ-GRB10 associated protection in IDD may involve ERBB2/Erk signaling.

### Key factors regulating circ-GRB10 formation

**Fig. 2:**
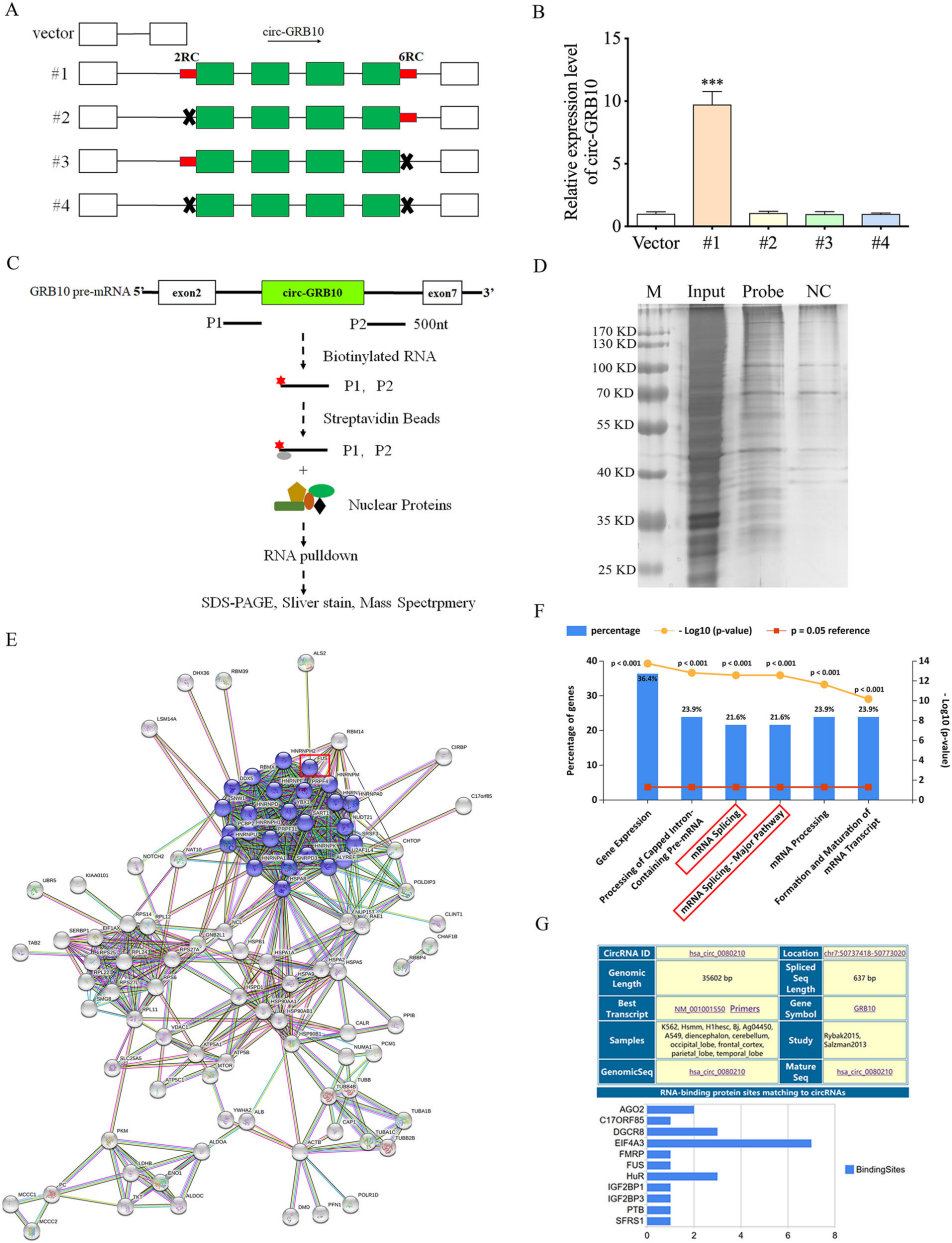
RBPs interact with GRB10 pre-mRNA. **a** A schematic drawing of four types of circ-GRB10 overexpressing vectors (#1 to #4). The genomic region for circ-GBR10 (green bars) with its wild-type flanking introns (black lines) was inserted into the pcDNA3.1 expression vector (#1). 2RC and 6RC are indicated by red bars. A series of deletions are indicated by black crosses (#2 to #4). **b** qRT-PCR showed the expression of circ-GBR10 after transfection with the four types of circ-GRB10 overexpressing vectors (#1 to #4). Three independent repeats were performed in each experiment. ***p < 0.001. **c** Schematic diagram of RNAs corresponding to different fragments of GRB10 pre-mRNA (P1, P2) produced by in vitro transcription in the presence of biotin for RNA pulldown experiments. **d** Silver stain acrylamide gel of total nuclear proteins before (Input) and after pulldown with biotin-labeled RNA probe (P1, P2). M, molecular weight marker (kDa). **e** Proteins identified from mass spectrometry were integrated to STRING database and constructed Protein-protein interaction (PPI) network. A densely connected module which contains 27 proteins, including FUS, was screened from the PPI network, and these proteins were participate in biological process of mRNA splicing, via spliceosome. **f** Pathways enrichment analysis of proteins in PPI network. **g** RBPs which can potentially bind circ-GRB10 pre-mRNA.

In this study, we found a highly reverse complementary sequence 500 nt upstream the 5′ splice site in intron 2 and one 500 nt downstream the 3′ splice site in intron 6, which were named 2RC (reverse complementary sequence in intron 2) and 6RC (reverse complementary sequence in intron 6), respectively. Then, wild-type (sequence spanning from intron 2 to intron 6 of the GRB10 gene, #1) and multiple deletion constructs (#2–4) for circ-GRB10 were introduced into pcDNA3.1(+), respectively (Fig. 2a). Upon transfection, the wild-type vector (#1), unlike the 2RC and/or 6RC deletion constructs (#2–4), overexpressed circ-GRB10, indicating 2RC and 6RC may contain the binding sites that regulate circ-GRB10 biogenesis (Fig. 2b).

As circRNAs are derived from pre-mRNAs, and circRNAs could be regulated by RBPs post transcriptionally^18,21,22,40^, we hypothesized that circ-GBR10 is modulated by RBPs post-transcriptionally in IDD development. To identify the RBPs which might regulate GRB10 pre-mRNA splicing to generate circ-GRB10, we incubated biotin labeled sequences (cloned from circ-GRB10 back splicing site 500 nt upstream (P1) or 500 nt downstream (P2)) with nuclear protein extracts from normal human NP cells (Fig. 2c). Nuclear proteins bound to RNA underwent separation by SDS-PAGE and silver staining (Fig. 2d), followed by mass spectrometry for identification. A total of 143 proteins (Supplementary Table S1) were retrieved and mapped to the STRING database, screening significant interactions with scores above 0.7 (Fig. 2e). Enrichment analysis demonstrated that these 143 proteins were mainly involved in the pathways of gene expression, processing of capped intron-containing pre-mRNA, mRNA splicing, mRNA splicing-major pathway, mRNA processing and formation and maturation of mRNA transcript related signaling pathways (Fig. 2f). Among these, 19 proteins were involved in the mRNA splicing and mRNA splicing-major pathway (Supplementary Table S2). In addition, the web tool CircInteractome predicted 11 RBPs which can potentially bind circ-GRB10 pre-mRNA (Fig. 2g). Notably, FUS was the only RBP that was involved in mRNA splicing and could potentially bind to circ-GRB10 pre-mRNA, suggesting circ-GRB10 generation may be associated with FUS expression in NP cells.

### FUS promotes the generation of circ-GRB10 in NP cells

**Fig. 3:**
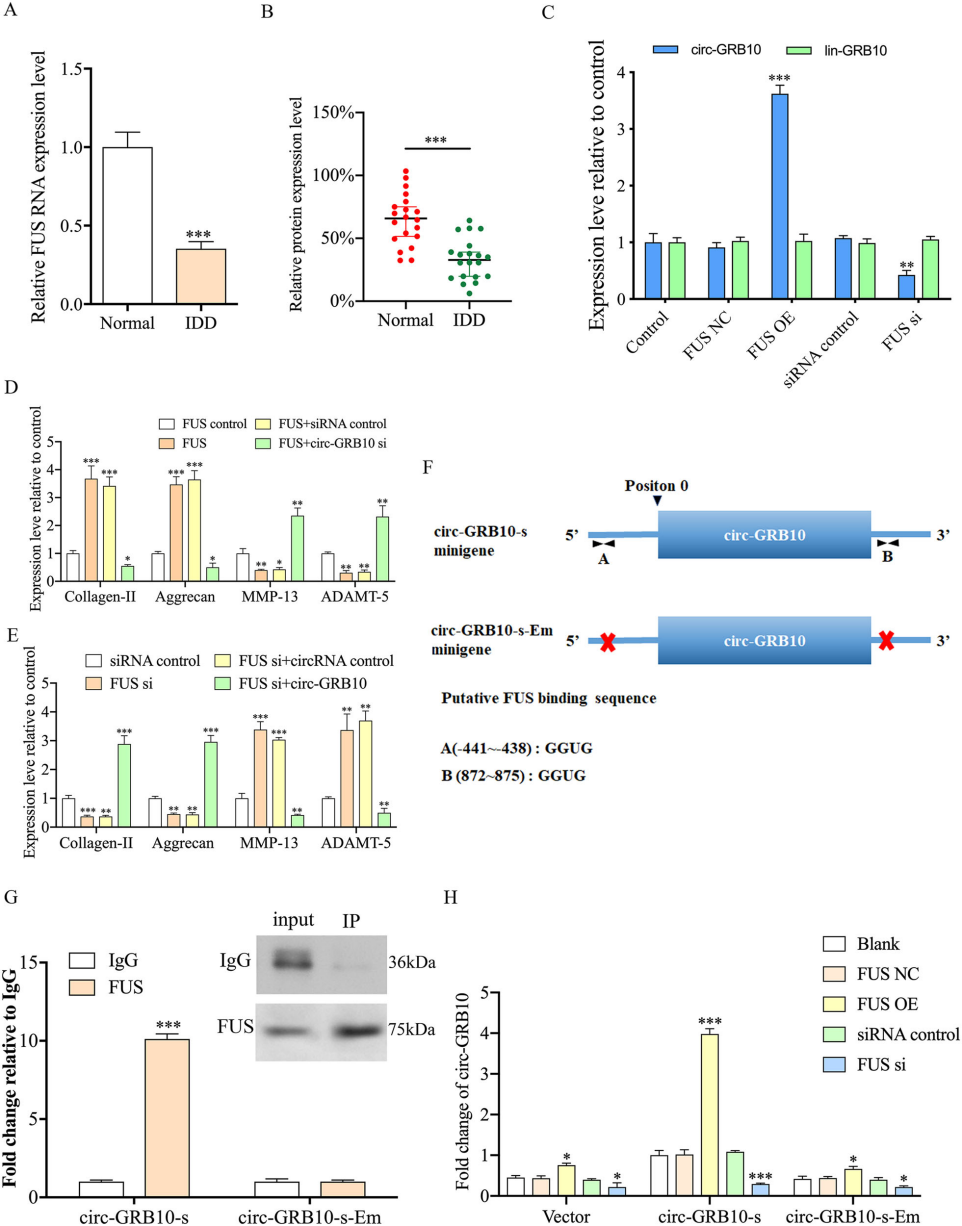
FUS regulates the generation of circ-GRB10 in NP cells. **a** qRT-PCR showing FUS mRNA levels in normal and IDD NP tissues. Three independent repeats were performed in each experiment. ***p < 0.001. **b** Western blot showing FUS protein amounts were decreased in IDD NP tissues. **c** qRT-PCR analysis of circ-GRB10 expression level after FUS overexpression or knockdown in NP cells. FUS overexpression led to significantly increased circ-GRB10 amounts in NP cells, while its knockdown reduced circ-GRB10 levels. Moreover, FUS had no linear effects on GRB10 expression. Three independent repeats were performed in each experiment. ***p < 0.001. **d**, **e** qRT-PCR analysis of the expression of Collagen-II, aggrecan, MMP-13 and ADAMT-5 in NP cells. **f** Schematic illustrating the putative FUS-binding sites on the flanking introns in the circ-GRB10-s minigene. The 5′ terminus of the circular exons of circ-GRB10 was defined as position 0. Putative FUS-binding sites A and B are located in the intron at the 5′ terminus of the circ-GRB10 exon (position: −441 to −438), and on the intron at the 3′ terminus of the circ-GRB10 exon (position: 872–875). **g** RIP analysis of FUS-binding to circ-GRB10-s and circGRB10-s-Em minigenes in NP cells. Bound complexes were pulled-down using an antibody against FUS. qRT-PCR was then used to measure circ-GRB10-s binding to FUS. Values were normalized to the level of background RIP, as detected by an IgG isotype control. **h** qRT-PCR analysis of the expression of circ-GRB10 relative to GAPDH in NP cells. Cells were co-transfected with FUS or FUS siRNA and a circ-GRB10 minigene (circ-GRB10-s), or circ-GRB10 minigene containing deleted FUS-binding sites (circ-GRB10-Em). Quantitative data from three independent experiments is presented as mean ± SEM (error bars). **P < 0.01; ***P < 0.001.

Recently, FUS was reported to have a role in regulating circRNA biosynthesis via binding of introns surrounding the back-splicing junctions^31^. As shown in Fig. 3A, FUS amounts in IDD NP tissues were remarkably lower than those of controls. In addition, Western blot further confirmed this result (Figs. 3b and S2). To assess whether FUS contributes to circ-GRB10 production in NP cells, we overexpressed or suppressed FUS, and determined circ-GRB10 amounts. qRT-PCR demonstrated that FUS overexpression led to significantly increased circ-GRB10 amounts in NP cells while FUS knockdown reduced the expression of circ-GRB10 (Fig. 3c). Moreover, FUS had no effects on linear GRB10 expression (Fig. 3c). Overexpression of FUS resulted in increased collagen-II and aggrecan amounts, and decreased MMP-13 and ADAMT-5 levels in NP cells, while the circ-GRB10 siRNA attenuated these changes (Fig. 3d). FUS knockdown resulted in downregulated collagen-II and aggrecan, and upregulated MMP-13 and ADAMT-5 in NP cells, while circ-GRB10 markedly counteracted the effects of FUS knockdown, indicating that FUS exerted its functions through circ-GRB10 (Fig. 3e).

To assess whether FUS-binding sequences are important in circ-GRB10 biosynthesis, FUS-binding sequences were searched in circ-GRB10 and surrounding introns, and two putative FUS-binding sites were detected (Fig. 3f). Next, two short circ-GRB10 minigenes were engineered, including circ-GRB10-s and circ-GRB10-s-Em. Precisely, circ-GRB10-s comprises presumed FUS-binging sites on both flanking introns preserved, with the inversely inserted 5′ intron in circ-GRB10 removed to prevent complementary sequences from reacting (Fig. 3f); circ-GRB10-s-Em resembles circ-GRB10-s, but with FUS sites deleted from the surrounding introns (Fig. 3f). RIP revealed an overt interaction of FUS with circ-GRB10-s, unlike circB-s-Em (Fig. 3g), indicating FUS required the putative sites in surrounding introns for binding. We next expressed circ-GBR10-s in FUS overexpressing or knocked down NP cells, and circ-GRB10-s yielded elevated amounts of circ-GRB10 transcript after FUS overexpression and reduced amounts upon FUS knockdown, confirming FUS is important in circ-GRB10 biosynthesis in NP cells (Fig. 3h). Next, circ-GRB10-s-Em was expressed in NP cells, and it yielded markedly reduced circ-GRB10 amounts in comparison with circ-GRB10-s (Fig. 3h). This indicated that the putative FUS-binding sequences in the surrounding introns were important in circ-GRB10 biosynthesis. Taken together, the above findings demonstrated that FUS had a critical regulatory function in circ-GRB10 biosynthesis in NP cells via binding to recognition sites in the introns surrounding the circ-GRB10-forming exons.

### FUS in NP cells is regulated by miR-141-3p

**Fig. 4:**
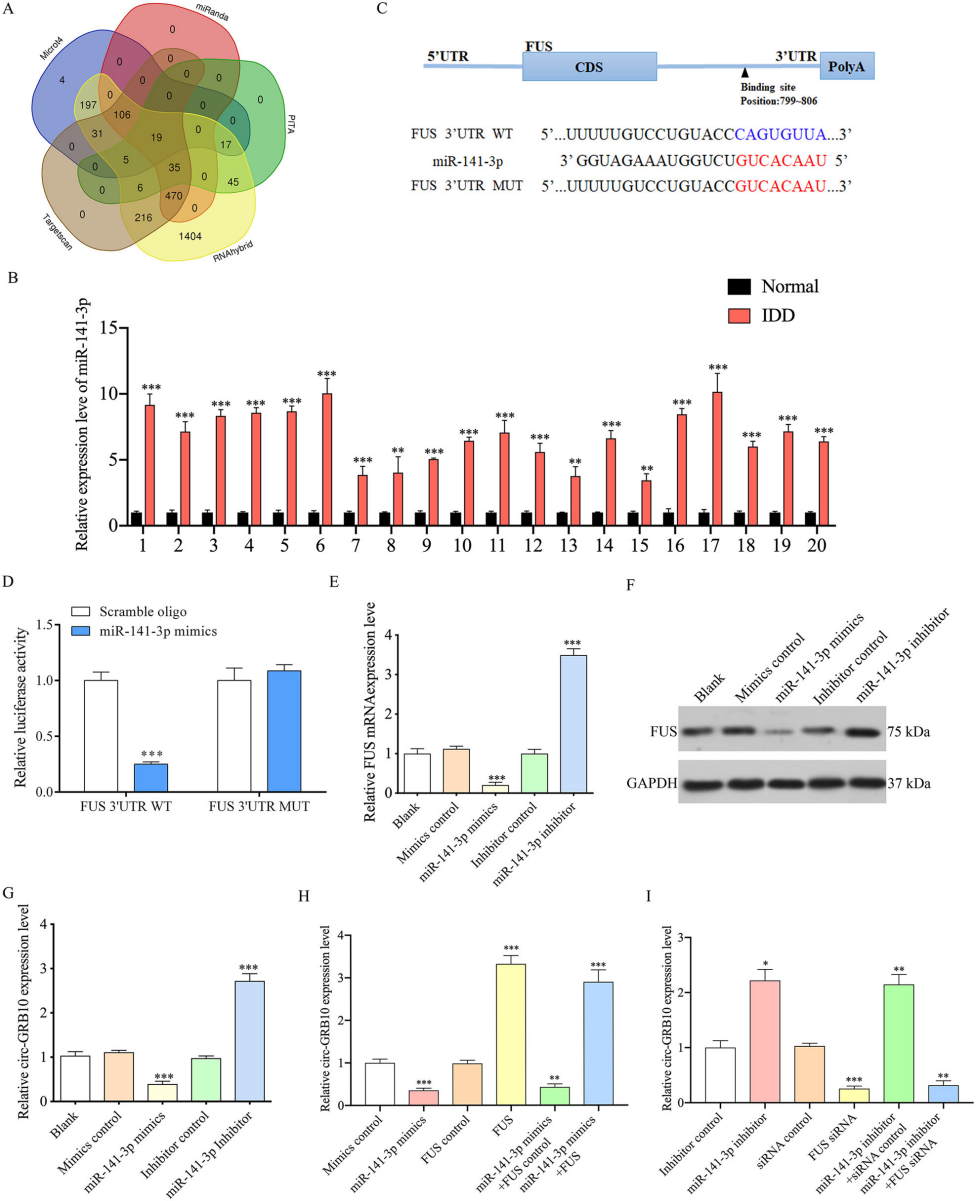
miR-141-3p inhibits FUS expression in NP cells. **a** The Venn diagram indicates the 19 predicted miRNAs regulate FUS expression. miR-141-3p was intersected predicted by 5 different databases. **b** Expression of miR-141-3p in IDD NP tissues, showing that miR-141-3p expression was significantly higher than that of controls. Quantitative data from three independent experiments is presented as mean ± SEM (error bars). ***P < 0.001. **c** Sequence alignment of a putative miR-141-3p-binding site within the 3′UTR of FUS mRNA. Bottom: mutations in the 3′UTR of FUS mRNA sequence to create the mutant luciferase reporter constructs. **d** Luciferase reporter assay in NP cells after transfected with scramble oligo or miR-141-3p mimics, Renilla luciferase vector, and the reporter constructs. Both firefly and Renilla luciferase activities are measured in the same sample. Firefly luciferase signals were normalized with Renilla luciferase signals. Quantitative data from three independent experiments is presented as mean ± SEM (error bars). ***P < 0.001. e, **f** FUS expression level was detected by qRT-PCR, western blot in primary human NP cells. Three independent experiments is presented as mean ± SEM (error bars). ***P < 0.001. **g** NP cells from control tissues were transfected with miR-141-3p mimic or miR-141-3p inhibitor. qRT-PCR was used to detect the relative expression level of circ-GRB10 compared with controls. Three independent experiments is presented as mean ± SEM (error bars). ***P < 0.001. **h** NP cells from control tissues were transfected with miR-141-3p with or without FUS overexpress plasmid. qRT-PCR was used to detect the relative expression level of circ-GRB10 compared with controls. **i** miR-141-3p inhibitor with or without FUS siRNA was transfected into NP cells from control tissues and the expression level of FUS. Three independent experiments is presented as mean ± SEM (error bars). ***P < 0.001.

The mechanism of FUS downregulation in NP cells of IDD patients remains unclear. Previous studies have demonstrated that FUS is regulated by miRNAs in many deseases^32,33^. Therefore, we hypothesized that FUS may be regulated by miRNAs in NP cells. Using the Targetscan, Microt4, miRanda, PITA, and RNAhybird databases, all predicted miRNAs were retrieved and submitted to Venn analysis (Fig. 4a). The results showed that FUS was predicted to be regulated by 19 miRNAs (Supplementary Table S3), including miR-141-3p. Svetoni et al confirmed that miR-141-3p regulates FUS expression during neural differentiation and Ji et al. revealed miR-141-3p is associated with disc degeneration^33,34^. Furthermore, qRT-PCR showed that miR-141-3p was markedly upregulated in NP tissue samples from IDD cases in comparison with control values (Fig. 4b). Therefore, we supposed that FUS expression was regulated by miR-141-3p in NP cells. To further assess miR-141-3p interaction with FUS, luciferase reporter assays were carried out. Co-transfection of FUS WT (wild type) and miR-141-3p mimic in primary human NP cells resulted in markedly decreased luciferase activity in comparison with the FUS-mut (mutant)/miR-141-3p mimic co-transfection group (Fig. 4c, d). These findings were further confirmed at the gene and protein levels in human NP cells in vitro (Fig. 4e, f), pointing to FUS as a miR-141-3p target. Then, primary human NP cells underwent transfection with miR-141-3p mimic and miR-141-3p inhibitor, and the corresponding negative controls, respectively. The results showed that circ-GBR10 was significantly downregulated in cells overexpressing miR-141-3p (Fig. 4g). Conversely, circ-GRB10 was upregulated in the miR-141-3p inhibitor group (Fig. 4g). Moreover, upregulation of FUS alleviated the suppressive effects of miR-141-3p on circ-GRB10 expression (Fig. 4h), while FUS knockdown attenuated the effects of miR-141-3p inhibitor on circ-GRB10 upregulation (Fig. 4i). The above results indicated that miR-141-3p regulates circ-GRB10 expression in NP cells primarily through targeting of FUS.

### ERBB2 regulates miR-141-3p in NP cells by phosphorylating Erk1/2

**Fig. 5:**
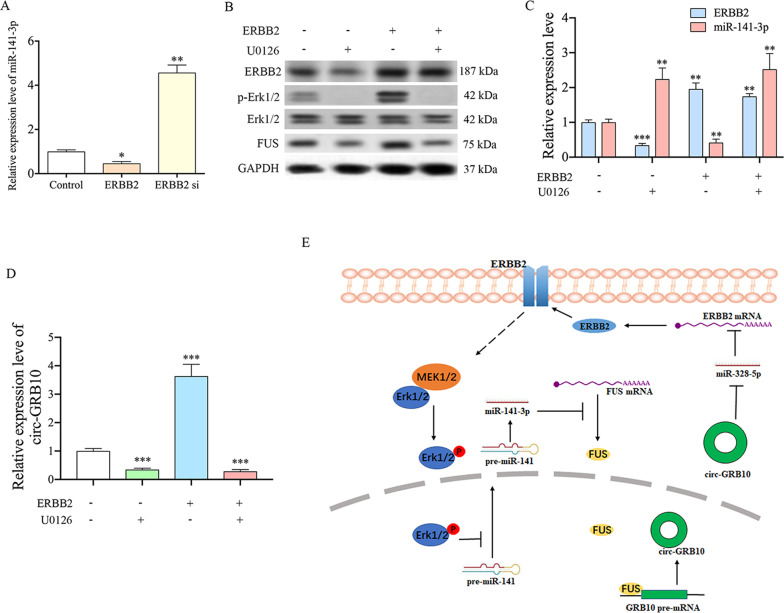
ERBB2 regulate miR-141-3p expression in NP cells. **a** miR-141-3p expression level in NP cells with ERBB2 overexpression or ERBB2 knockdown. Three independent experiments is presented as mean ± SEM (error bars). **P < 0.01. **b** NP cells overexpressing ERBB2 were treated with U0126 or not for one hour. Western blot was used to detect the phosphorylated level of Erk1/2. **c**, **d** NP cells overexpressing ERBB2 were treated with U0126 or not. qRT-PCR was used to detect the expression level of miR-141-3p and circ-GRB10. Three independent experiments are presented as mean ± SEM (error bars). **P < 0.01. **e** Schematic representation of mechanisms by which circ-GRB10 mediates IDD development. On the basis of findings described in the manuscript, miR-141-3p downregulates FUS level in NP cells, leading to circ-GRB10 decreased. This down-regulated circ-GRB10, in turn, enhanced miR-328-5p mediated suppression of ERBB2 in NP cells, leads to decreased Erk1/2 phosphorylation level. And the decreased Erk1/2 phosphorylation level enhances the generation of miR-141-3p in NP cells.

Induced Erk1/2 signaling causes widespread miRNA repression via suppression of the main steps of miRNA biogenesis^41,42^. In this study, we found decreased levels of Erk1/2 phosphorylation in circ-GRB10 or ERBB2 knocked down NP cells (Fig. 1d, e). Previous studies demonstrated that phosphorylated Erk1/2 can cause wide-spread miRNA repression through suppressing the major steps of miRNA biogenesis^41–43^. As ERBB2 amounts were reduced in degenerative NP cells, we hypothesized that miR-141-3p may be regulated by Erk1/2 phosphorylation in NP cells. To explore this possibility, we overexpressed or knocked down ERBB2 in NP cells. qRT-PCR results demonstrated that overexpression of ERBB2 significantly downregulated miR-141-3p, while ERBB2 knockdown increased miR-141-3p amounts (Fig. 5a). In addition, pre-treatment of NP cells with the Erk1/2 phosphorylation inhibitor U0126 downregulated ERBB2 and attenuated ERBB2 induced phosphorylation of Erk1/2, decreasing the expression of FUS (Fig. 5b). Moreover, blocking Erk1/2 phosphorylation in NP cells significantly attenuated ERBB2’s effects on miR-141-3p biogenesis (Fig. 5c) and decrease the expression of circ-GRB10 (Fig. 5d). Collectively, the above findings demonstrated that ERBB2 regulated miR-141-3p expression in NP cells by phosphorylating Erk1/2.

Next, we found that decreased ERBB2 amounts in degenerative NP cells could promote miR-141-3p generation which suppressed the expression of FUS, resulting in circ-GRB10 downregulation; our previous study demonstrated that circ-GRB10 downregulation leads to ERBB2 reduction by enhancing miR-328-5p mediated suppression of ERBB2 in NP cells^15^. These findings suggested circ-GBR10 contributed to the molecular circuitry controlling IDD development in humans (Fig. 5e).

### Intradiscal injection of circ-GRB10 alleviates IDD in a rat model

**Fig. 6:**
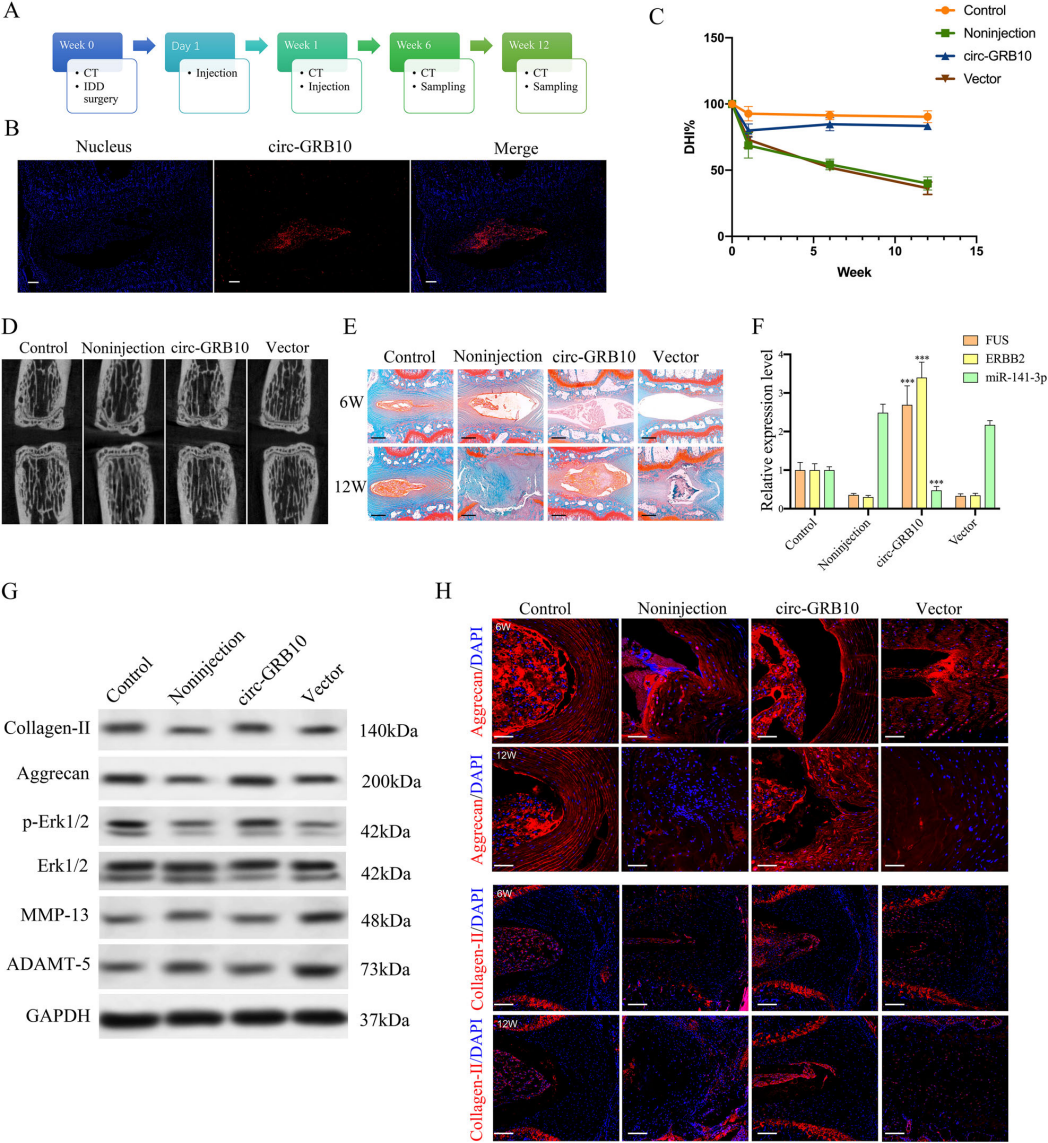
cric-GRB10 attenuates IDD development in vivo. **a** Overview of the experimental set-up with injections of circ-GRB10, or their negative control at 1, and 7 days after surgery. **b** Six weeks after surgery, in vivo RNA FISH found circ-GRB10 located in the NP region. Blue fluorescence (4,6-diamidino-2-phenylindole, DAPI) indicating cell nucleus; Red fluorescence indicating circ-GRB10. Scale bar = 200 μm. **c** Changes in disc height index (DHI) of the indicated groups after needle puncture. The DHI was measured at 0, 1, 6, and 12 weeks. A significant decrease of the DHI% was observed in all puncture groups at 1 week after surgery (*P* < 0.01). A significant increase in DHI% was noted at 6 and 12 weeks post surgery in rat treated with circ-GBR10 (*P* < 0.001). **d** CT scan of the indicated groups were obtained 12 weeks after needle puncture. **e** The intervertebral disc degeneration evaluated by Safranin O staining. Scale bar = 500 µm. **f** qRT-PCR showed that the increased levels of FUS, ERBB2 and the decreased level of miR-141-3p in the punctured discs treated with circ-GRB10. Three independent experiments are presented as mean ± SEM (error bars). ***P < 0.001. **g** Western blot showing the expression levels of collagen II, aggrecan, p-Erk1/2, MMP13, ADAMT-5 in rat NP tissues. **h** Immunostaining for collagen-II and aggrecan in IDD model treated by circ-GRB10 at 6 and 12 weeks. Scale bar = 100 µm.

Needle puncture has been one of the most common methods to establish animal models of IDD^44,45^. We have successfully established a rat model of IDD by needle puncture according to the above methods in this study (Supplementary Fig. S3). At 1 and 7 days upon modeling, adenoviral human circ-GRB10 was injected into the punctured intervertebral discs with 33-G needles. In vivo RNA FISH indicated circ-GRB10 in the NP region at 6 weeks after surgery (Fig. 6b). CT scan at 0, 1, 6, and 12 weeks revealed progressive disc space narrowing in all punctured animals and a significant increase in DHI% was noted at 6 and 12 weeks post surgery in rats treated by circ-GRB10 (Fig. 6c). CT scan revealed that overexpression of circ-GRB10 markedly preserved the progressive disc space narrowing in rat IDD model(Fig. 6d). And safranin O staining results demonstrated that overexpression of circ-GRB10 can inhibit the degradation of extracellular matrix of NP cells (Fig. 6e); These results suggesting circ-GRB10 exerted protective effects in surgically induced IDD. After injection of adenoviral circ-GBR10, FUS and ERBB2 amounts in degenerative NP tissues were remarkably elevated (Fig. 6f), while miR-141-3p amounts were decreased (Fig. 6f). In addition, injection of adenoviral circ-GBR10 alleviated the degenerative alterations of the NP, including enhanced Erk1/2 phosphorylation, collagen-II and aggrecan upregulation and inhibited the expression of MMP-13, ADAMT-5 in the rat model of IDD (Fig. 6g). Moreover, Immunofluorescence staining also confirmed that the increased expression of collagen-II. aggrecan and the downregulation of MMP-13, ADAMT-5 expression in the circ-GRB10 group compared with non-injection group at 12 weeks (Fig. 6h). Jointly, the above findings suggested a therapeutic role for circ-GRB10 in protecting the discs, revealing circ-GRB10 as a candidate therapeutic target in IDD.

## Discussion

Numerous circRNAs are found in the human transcriptome, playing critical roles in the regulation of cell functions^17,46,47^. Our previous study showed that circ-GRB10 downregulation is associated with human NP cell apoptosis^15^. However, the mechanism of circ-GRB10 dysregulation in IDD has not been previously described. Here, we found that FUS regulated and promoted circ-GRB10 biosynthesis by interacting with its flanking introns. In addition, FUS expression in NP cell was regulated by miR-141-3p. Our findings suggest a regulatory network in NP cells: FUS bound to GRB10 pre-mRNA to regulate circ-GRB10 synthesis, while circ-GRB10 acted as a molecular sponge for miR-328-5p, with suppressive effects on ERBB2 production and modulated IDD development; downregulation of ERBB2 decreased Erk1/2 phosphorylation and promoted the generation of miR-141-3p, which bound to the 3′UTR region of FUS to inhibit its expression, constituting a positive feedback loop promoting intervertebral disc degeneration.

The differential expression of circ-GBR10 between normal and degenerative NP tissues indicates that circ-GRB10 biosynthesis is controlled differently in various cells, with NP cells possessing specific factors required for circRNA biogenesis. Indeed, introns 2 and 6 of the GRB10 pre-mRNA had binding sites to regulate circ-GRB10 biogenesis. Furthermore, multiple RBPs were highly enriched in circ-GRB10’s flanking introns, and FUS contributed to circ-GRB10 biogenesis, as shown above. Although FUS induced circ-GRB10 biosynthesis, its silencing only decreased circ-GRB10 levels by ~50%, as shown above. It is known that two or more RBPs could control the synthesis of circRNAs synergistically^40,48^, which might explain the above incomplete suppression. Therefore, circ-GRB10 modulation in NP cells deserves further examination.

As shown above, altered FUS expression might profoundly affect circ-GRB10 biogenesis. Further, deletion of FUS-binding sequences in the introns flanking of circ-GBR10 dramatically reduced circ-GRB10 amounts. Taken together, the above findings indicate FUS may directly control back-splicing to upregulate circ-GRB10 in NP cells by interacting with putative binding sequences on both flanking introns of circ-GRB10.

Recently, miR-141 has been detected in NP tissue samples from IDD cases, and its levels correlate with disc degeneration. Therefore, miR-141 NPs have been used in IDD treatment with commendable efficacy^34^. As shown above, miR-141-3p, which is a key regulator of IDD, directly regulated FUS, further revealing the FUS/circ-GRB10 axis as an IDD-related regulatory pathway.

Accumulating evidence indicates that Erk signaling has an important role in IDD^39,49,50^. In the present study, we found that circ-GRB10 significantly upregulated collagen II and aggrecan in NP cells, and mediated the protective effects in IDD likely via ERBB2/Erk signaling. There might also be cellular pathways that compensate for ERBB2 expression after its knockdown. For example, the long intergenic noncoding RNA (lincRNA) BCLIN25 upregulates ERBB2 by inducing promoter CpG methylation of miR-125b, resulting in miR-125b repression^44^. A previous study indicated the Erk pathway regulates the miRNA-generating complex^43^. In addition, Sun et al. found that Erk1/2 activation results in global miRNA downregulation^41^. As shown above, Erk1/2 phosphorylation levels were significantly decreased upon ERBB2 downregulation in NP cells. Furthermore, U0126 could block the ERBB2 regulatory effects on miR-141-3p. These results, at least in part, demonstrate that miR-141-3p is significantly upregulated in degenerative NP cells due to ERBB2 downregulation. However, future studies are required to elucidate how Erk1/2 regulates miR-141-3p generation in NP cells.

Moreover, in this study, we demonstrated that circ-GRB10 expresson level is important for NP cells function. More importantly, circ-GRB10 was applied as a highly effective method for IDD treatment in rat model. These findings indicate the potential use of circ-GRB10 as a promising therapeutic tool for treating IDD.

Overall, our study revealed that the circ-GRB10 suppresses IDD development by alleviating NP cells imbalance between anabolism and catabolism of ECM and may serve as a potential therapeutic target in IDD. In addition, a regulatory role for the ERBB2/Erk1/2/miR-141-3p/FUS/circ-GRB10/miR-328-5p/ERBB2 network was preliminarily confirmed in NP cells. Our findings demonstrated that circ-GBR10 contributes to the molecular circuitry controlling IDD development in humans, providing a basis for further functional, diagnostic and therapeutic assessments of circ-GRB10 in IDD.

## Materials and methods

### Patient specimens

Human lumbar degenerative NP samples were from 20 IDD cases undergoing discectomy (12 males and 8 females; age 34.95 ± 5.10 years). Control specimens were obtained from 18 age- and sex-matched individuals suffering from fresh traumatic lumbar fractures undergoing anterior decompressive surgery due to neurological impairment (13 males and 7 females; age 33.83 ± 5.51 years). Based on Pfirrmann classification, disc degeneration was assessed on T2-weighted images^51^. The present study had approval from the ethics committees of Tianjin Medical University General Hospital and Hebei Province Cangzhou Hospital of Integrated Traditional and Western Medicine.

### NP tissue harvest, and NP cell isolation and culture

NP tissue samples were separated, cut into pieces, and incubated in presence of 0.25% pronase (Sigma, USA; 30 min) and 0.2% collagenase type II (Invitrogen, USA; 4 h) at 37°C. The digested samples underwent filtration through a 70 μm pore mesh and culture in Dulbecco’s Modified Eagle’s Medium (DMEM; Gibco, USA) containing 10% fetal bovine serum (FBS; Invitrogen), 1% penicillin–streptomycin (Sigma), 2 mM glutamine (Sigma), and 50 μg/mL l-ascorbic acid (Sigma), at 37 °C in presence of 5% CO2. At confluence, cells were passaged upon digestion with 0.25% trypsin/1 mM EDTA, and assessed at passages 3–5.

### Immunofluorescence

Human NP cells grown on cover glass underwent fixation with 4% formalin (20 min) at ambient, permeabilization with 0.1% Triton X-100 and 0.2% Tween-20 in PBS (40 min at ambient), blocking with 2% goat serum (Invitrogen; 1 h) and incubation with anti-collagen-II (1:200; Abcam, Ab34712), anti-Aggrecan (1:500; Abcam, Ab5790), anti-MMP13 (1:50; Abcam, Ab21624), and anti-ADAMT-5 (1:1000; Millipore, MAB4401) primary antibodies, respectively. After washing, the samples further underwent incubation with fluorescein-conjugated secondary antibodies. Images were captured under a fluorescence microscope (Leica). Relative fluorescence intensity was calculated with Image J.

### Cell transfection

Passage 3 NP cells were transfected with respective plasmids or siRNAs with Lipofectamine 3000 (Invitrogen) as described previously^52^ for 48 h.

### Western blot

Samples underwent lysis, and protein amounts in the lysates were assessed with Micro BCA Protein Assay Kit (Thermo, USA). Cell lysis was carried out in a buffer comprising 0.25 M Tris-HCl, 20% glycerol, 4% sodium dodecyl sulfate (SDS), and 10% mercaptoethanol (pH 6.8) with protease and phosphatase inhibitors. Equal amounts of total protein (10 µg) underwent separation by 10–12% SDS-PAGE and electro-transfer onto polyvinylidene fluoride membranes. Then, 5% skim milk in Tris-buffered saline containing 0.1% Tween-20 (TBST) was employed for blocking at ambient (1 h), followed by incubation with primary antibodies in TBST containing 5% non-fat milk overnight at 4 °C. Secondary antibodies (1:6000) were added at room temperature for 1 h, and development was performed with the enhanced chemiluminescence system. The primary antibodies tested targeted collagen-II (1:1000; ab34712, Abcam), aggrecan (1:500; ab194594, Abcam), MMP-13 (1:3000; Abcam ab39012,), ADAMTS-5 (1:250; Abcam ab41037,), Erk1/2 (1:1000; Abcam ab17942), p-Erk1/2 (1:300; Abcam ab214362) and FUS (1:10000; Abcam ab70381). The antibody targeting GAPDH (ab9485, 1:2500; Abcam) was assessed as an internal control.

### Quantitative real-time PCR (qRT-PCR)

M-MLV reverse transcriptase (Invitrogen) was employed for reverse transcription of total RNA as directed by the manufacturer. The mRNA levels were assessed by SYBR Green-based qPCR. PCR amplification was carried out in 10-μL reactions comprising cDNA (2 μL), 2× master mix (5 μL), forward and reverse primers (10 μM; 0.5 μL), and water (2 μL) at 95 °C (10 min), followed by 40 cycles of 95 °C (10 s) and 60 °C (60 s). Sample analysis was performed by the 2^−ΔΔCt^ method^53^, with GAPDH as a reference gene. Meanwhile, miRNA amounts were quantified with the stem-loop miRNA RT-PCR Quantitation kit (GenePharma). For circRNA detection, total RNA samples were treated with or without 3 U/μg of RNase R (Epicentre, USA) at 37 °C for 20 min, and the resulting RNA subsequently underwent purification with RNeasy MinElute Cleanup Kit (Qiagen). Specific divergent primers for the back-splice junction of circ-GRB10 were used to amplify the circRNA. The resulting amplification products were detected by agarose gel electrophoresis and sequencing. U6 and GAPDH served as references for miRNAs and circRNAs, respectively. All primers are listed in Supplementary Table S4.

### Subcellular fractionation

NP cells underwent lysis with a buffer comprising 50 mM KCl, 25 mM HEPES (pH 7.8), 1 mM phenylmethylsulfonyl fluoride (PMSF), 10 μg/mL leupetin, 25 μg/mL aprotinin, 100 μM dithiothreitol (DTT) and 0.5% NP-40. The lysates underwent centrifugation (2700 × *g*, 5 min; 4 °C), and supernatants were collected (cytosolic fraction). The precipitate was treated with chilled nuclear extraction buffer (500 mM KCl, 1.5 mM MgCl2, 25 mM HEPES, 1 mM PMSF, 10 μg/mL leupeptin, 25 μg/mL aprotinin, 100 μM DTT and 10% glycerol) for 30 min and centrifuged (20,000 × *g*, 10 min; 4 °C) for nuclear fraction collection.

#### Expression vectors

For circRNA-overexpressing FUW vectors, full-length circ-GRB10 lacking reacting introns were cloned from a cDNA library of NP cells. The endogenous 5′- and 3′-flanking 1 kb introns were cloned from the genomic DNA and ligated to the respective circ-GRB10 ends. Importantly, the exon–intron limits and canonical splicing sites (GT-AG) at both circ-GRB10’s termini were maintained. The most upstream 1 kb sequence of the 5′-flanking intron was inversely inserted downstream of the 3′-flanking intron. To construct the circ-GRB10-s vector, the inversely inserted 5′-flanking intron at the circ-GRB10’s 3′ terminus was deleted with restriction enzymes. For the circ-GRB10-s-Em vector, the sequence between both FUS-binding sites at both termini of the circ-GRB10-s vector was PCR cloned and ligated into the FUW vector.

#### 3′-Untranslated region cloning and luciferase assay

To generate the WT FUS 3′UTR-Luc reporter plasmid (FUS 3′UTR), a portion of the FUS′ 3′-Untranslated region (UTR) comprising the putative miR-141-3p-binding site was submitted to PCR and cloned into psi-CHECKTM-2 (Promega) downstream of firefly luciferase using XhoI and NotI (Thermo). Constructs harboring mutations at the predicted miR-141-3p-binding site in WT FUS 3′UTR were generated by site-directed mutagenesis with Quick Change Lightning Site-Directed Mutagenesis Kit (Agilent Technologies, USA). PCR reactions comprised 0.7 μL of expand long-range enzyme mix (Roche, Germany), 10 μL of 5× expand long-range buffer, 100 ng of plasmid template, 100 nM of each primer, 3 μL of dimethyl sulfoxide, and 2.5 μL of dNTPs (10 mM). PCR was performed at 92 °C (30 s), 55 °C (1 min), 68 °C (10 min), and 68 °C (10 min). Then, 20 μL of PCR products underwent digestion with DpnI (37 °C, 1 h) and 10 μL was used to transform *Escherichia coli* DH5α for producing mutant constructs. For luciferase assays, primary human NP cells were plated at 3000 cells/well in 96-well plates, and co-transfected with WT- or mutated- FUS 3′UTR-Luc reporter plasmid and miR-141-3p with Lipofectamine for 48 h. Luciferase activities were determined with a Dual-Glo Luciferase Assay system (Promega) as directed by the manufacturer.

#### RNA pull-down assay, silver staining, and mass spectrometry

Nuclear extracts (~300 μg) were added to denatured RNA (8 μg) corresponding to biotinylated circ-GRB10 flanking introns’ anti-sense, and submitted to overnight incubation at 4°C with yeast tRNA (Sigma) pre-treated streptavidin beads (Invitrogen). Bead collection was performed by centrifugation (20,000 × *g*, 1 min; 4 °C), and proteins were resolved by SDS-PAGE and submitted to silver staining (Pierce). Then, the immunoprecipitated proteins were resolved by SDS-PAGE, and bands were trypsin digested. The resulting peptides were assessed on a tandem time-of-flight mass spectrometer (4700 Proteomics Analyzer; Applied Biosystems). The Mascot, SEQUEST or Protein Prospector software was used for searching in the SwissProt database.

#### RNA immunoprecipitation

RNA immunoprecipitation (RIP) was carried out with Magna RIP Kit (Millipore) as directed by the manufacturer. Briefly, 2 × 10^7^ NP cells underwent UV-crosslinking at 600 mJ/cm^2^ and lysis with 100 μl RIP lysis buffer with a proteinase inhibitor cocktail (Roche) and RNase inhibitor (Promega). Lysates were incubated with DNase I (Roche) at 37 °C for 10 min and submitted to centrifugation at 12,000 × *g* for 30 min. The lysates were next mixed with 900 μl RIP immunoprecipitation buffer and treated for 3 h with 5 μg anti-FUS (Abcam, ab70381) antibodies pre-bound on magnetic beads. An aliquot (10 μl) of this RIP mixture was assessed in parallel. Bead washing (six times) was carried out with RIP wash buffer. Then, 20% of the immunoprecipitate was assessed by immunoblot and the remaining 80% underwent proteinase K treatment at 37 °C for 30 min. RNA extraction was carried out with TRIzol reagent (Invitrogen) as directed by the manufacturer.

#### The rat model of IDD

In this study, 48 male Sprague–Dawley rats (3 months) were assessed, adopting the IDD model^44,45^. In all, 36 rats underwent the surgery and the remaining 12 animals not operated constituted the negative control group. The animals were operated in the prone position following anesthesia (90 mg/kg ketamine and 10 mg/kg xylazine administered intraperitoneally). Under fluoroscopy, 3 intervertebral discs (Co6/7, Co8/9 and Co10/11) underwent puncture with 20G needles; Co7/8 and Co9/10 were untouched as controls. Standard postoperative procedures were carried out.

#### Intradiscal injection of circ-GBR10

One day following the initial intervertebral disc puncture, the rats were randomized into three groups (non-injection, circ-GRB10 injection, and circ-GRB10-mut injection) with 12 rats/group. After anesthesia, a small incision was made to expose the previously punctured intervertebral disc from the left side. A total of 2 μl solution containing the experimental or control virus vector (approximately 10^6^ plaque-forming units) overexpressing circ-GRB10 was slowly injected into the punctured discs with a 33G needle (Hamilton, Switzerland) attached to a microliter syringe (Hamilton). The injection procedure was repeated at 7 days after IDD surgery.

### Statistical analysis

All experiments were repeated three times or more. Continuous data are mean±standard deviation (SD). Multiple groups were compared by one-way analysis of variance and the Cochran’s Q test. Group pairs were compared by Student’s *t*-test. Categorical data were assessed by the chi-squared test. Prism 7.0 (GraphPad Software, USA) and SPSS 22.0 (SPSS Inc., USA) were employed for all statistical analyses. *P < 0.05, **P < 0.01, and ***P < 0.001 were significance levels.

## Supplementary information

Supplementary Figure Legends

Supplementary Figure S1

Supplementary Figure S2

Supplementary Figure S3

Supplementary Table S1

Supplementary Table S2

Supplementary Table S3

Supplementary Table S4
