# Supplementary material for: The circular RNA circ-GRB10 participates in the molecular circuitry inhibiting human intervertebral disc degeneration
Source: Cell Death Dis. 2020 Aug 13;11(8):612. doi: 10.1038/s41419-020-02882-3 (PMC7426430; doi:10.1038/s41419-020-02882-3)
Supplement: Supplementary file 1 — Supplementary Figure Legends [file 41419_2020_2882_MOESM1_ESM.docx]

**Supplementary Figure Legends**

**Figure S1**. qRT-PCR showing the expression levels of circ-GRB10 and its linear isoform (lin-GRB10) after overexpression or knockdown of circ-GRB10. Three independent experiments are presented as mean ± SEM (error bars). ***P<0.001.

**Figure S2.** Western blot showing FUS protein expression in IDD NP tissues versus controls. Human lumbar degenerative NP samples from IDD cases and control patients (n=20 per group) were assessed by immunoblot. Immunoreactive bands for FUS and GAPDH (loading control) are shown.

**Figure S3.** H&E and Safranin O staining of intervertebral disc from 0W, 1W, 6W and 22W after intervertebral disc puncture. Scale bar = 800 μm.
