# Supplementary figures and images for: The circular RNA circ-GRB10 participates in the molecular circuitry inhibiting human intervertebral disc degeneration
Source: Cell Death Dis. 2020 Aug 13;11(8):612. doi: 10.1038/s41419-020-02882-3 (PMC7426430; doi:10.1038/s41419-020-02882-3)

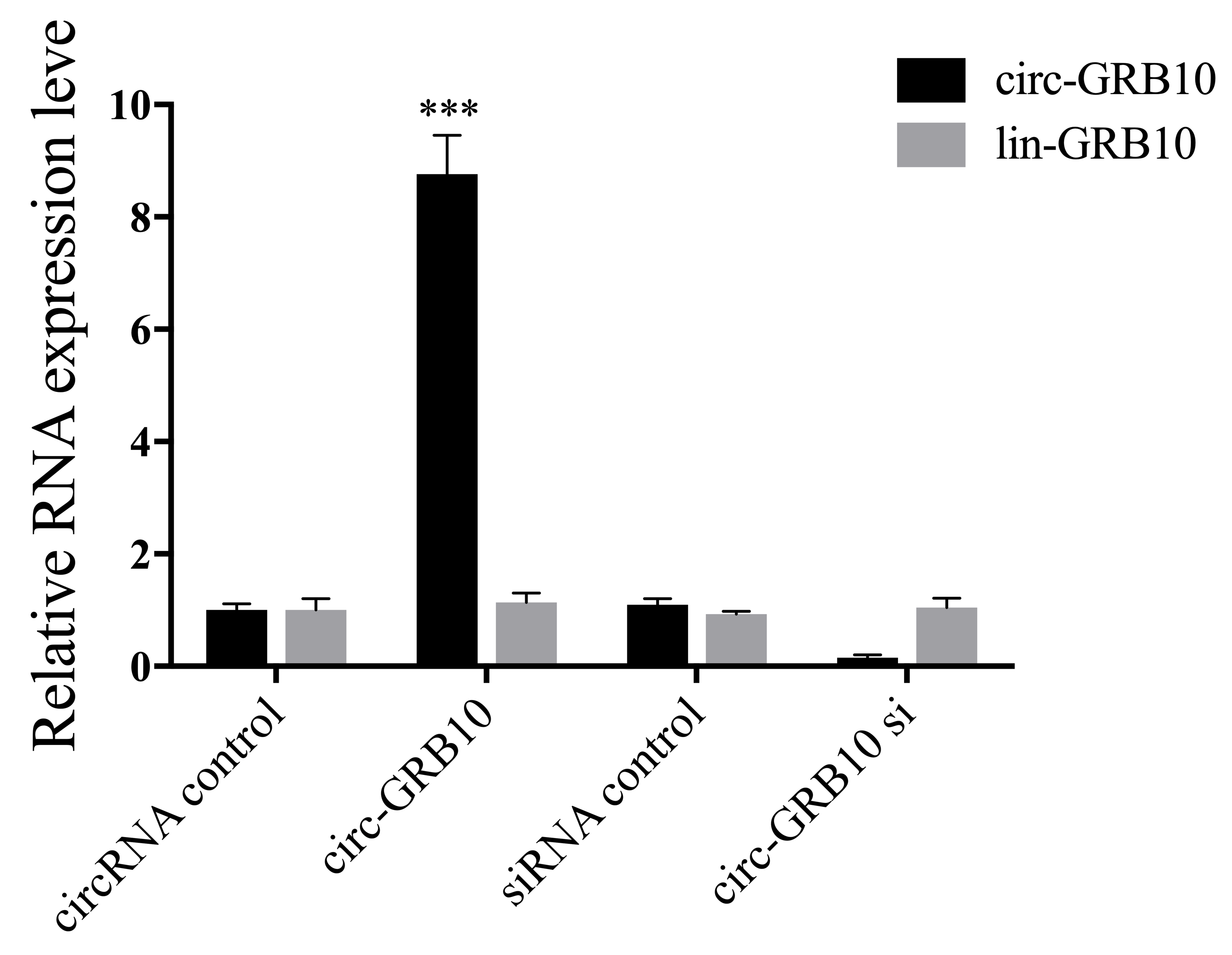

Supplement: Supplementary file 2 — Supplementary Figure S1 [file 41419_2020_2882_MOESM2_ESM.tif]

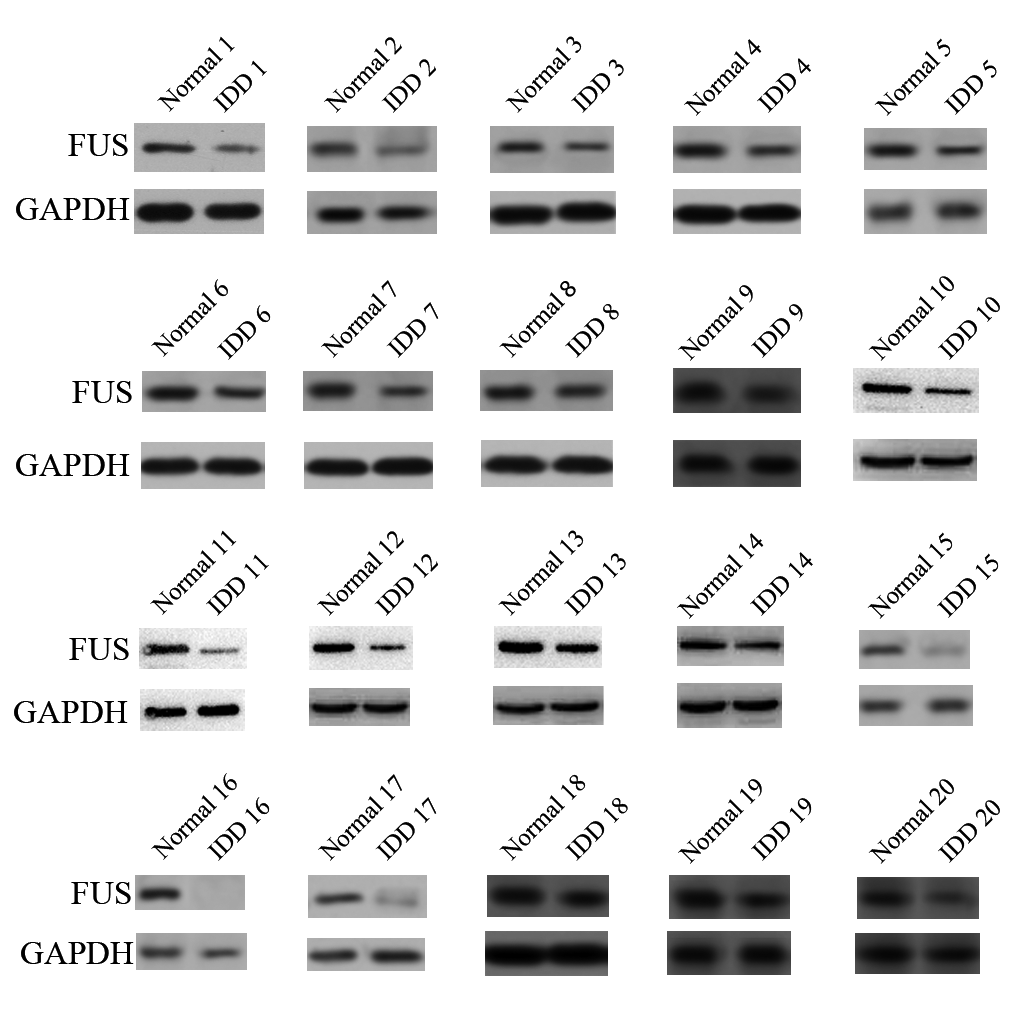

Supplement: Supplementary file 3 — Supplementary Figure S2 [file 41419_2020_2882_MOESM3_ESM.tif]

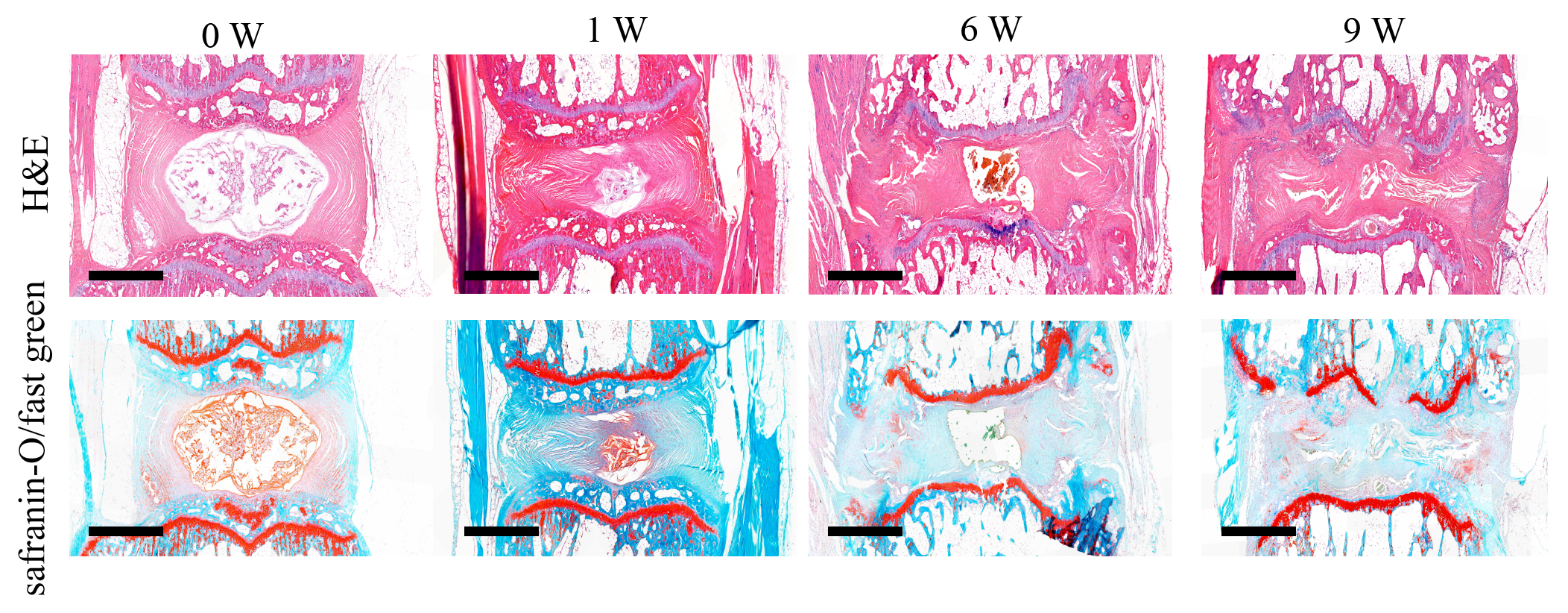

Supplement: Supplementary file 4 — Supplementary Figure S3 [file 41419_2020_2882_MOESM4_ESM.tif]
