# Supplementary material for: The circular RNA circ-GRB10 participates in the molecular circuitry inhibiting human intervertebral disc degeneration
Source: Cell Death Dis. 2020 Aug 13;11(8):612. doi: 10.1038/s41419-020-02882-3 (PMC7426430; doi:10.1038/s41419-020-02882-3)
Supplement: Supplementary file 5 — Supplementary Table S1 [file 41419_2020_2882_MOESM5_ESM.docx]

| Supplementary Table S1. proteins retrieved and mapped to the STRING database | | | | | | |
| --- | --- | --- | --- | --- | --- | --- |
| Accession | Score | Mass | Matches | emPAI | Protein description |  |
| [P52272](http://sale-depta-pc/mascot/cgi/master_results_2.pl?file=20181219%2FF002798.dat;pr.eh=3%2C3p;pr.page=3;pr.per_page=1;pr.show=proteins#tc:rf) | 634 | 77749 | 37 (27) | 1.02 | Heterogeneous nuclear ribonucleoprotein M OS=Homo sapiens OX=9606 GN=HNRNPM PE=1 SV=3 | |
| [P68363](http://sale-depta-pc/mascot/cgi/master_results_2.pl?file=20181219%2FF002798.dat;pr.eh=4%2C4p;pr.page=4;pr.per_page=1;pr.show=proteins#tc:rf) | 410 | 50804 | 22 (17) | 0.87 | Tubulin alpha-1B chain OS=Homo sapiens OX=9606 GN=TUBA1B PE=1 SV=1 | |
| [Q96PK6](http://sale-depta-pc/mascot/cgi/master_results_2.pl?file=20181219%2FF002798.dat;pr.eh=6%2C6p;pr.page=6;pr.per_page=1;pr.show=proteins#tc:rf) | 354 | 69620 | 18 (12) | 0.45 | RNA-binding protein 14 OS=Homo sapiens OX=9606 GN=RBM14 PE=1 SV=2 | |
| [P07437](http://sale-depta-pc/mascot/cgi/master_results_2.pl?file=20181219%2FF002798.dat;pr.eh=7%2C7p;pr.page=7;pr.per_page=1;pr.show=proteins#tc:rf) | 344 | 50095 | 17 (15) | 0.67 | Tubulin beta chain OS=Homo sapiens OX=9606 GN=TUBB PE=1 SV=2 | |
| P08238 | 272 | 83554 | 13 (8) | 0.31 | Heat shock protein HSP 90-beta OS=Homo sapiens OX=9606 GN=HSP90AB1 PE=1 SV=4 | |
| P11142 | 265 | 71082 | 15 (9) | 0.37 | Heat shock cognate 71 kDa protein OS=Homo sapiens OX=9606 GN=HSPA8 PE=1 SV=1 | |
| [P31943](http://sale-depta-pc/mascot/cgi/master_results_2.pl?file=20181219%2FF002798.dat;pr.eh=10%2C10p;pr.page=10;pr.per_page=1;pr.show=proteins#tc:rf) | 234 | 49484 | 8 (5) | 0.29 | Heterogeneous nuclear ribonucleoprotein H OS=Homo sapiens OX=9606 GN=HNRNPH1 PE=1 SV=4 | |
| [P68371](http://sale-depta-pc/mascot/cgi/master_results_2.pl?file=20181219%2FF002798.dat;pr.eh=7%2C7p;pr.page=7;pr.per_page=1;pr.show=proteins#tc:rf) | 232 | 50255 | 14 (12) | 0.46 | Tubulin beta-4B chain OS=Homo sapiens OX=9606 GN=TUBB4B PE=1 SV=1 | |
| [P35637](http://sale-depta-pc/mascot/cgi/master_results_2.pl?file=20181219%2FF002798.dat;pr.eh=11%2C11p;pr.page=11;pr.per_page=1;pr.show=proteins#tc:rf) | 230 | 53622 | 12 (10) | 0.35 | RNA-binding protein FUS OS=Homo sapiens OX=9606 GN=FUS PE=1 SV=1 | |
| [P09651](http://sale-depta-pc/mascot/cgi/master_results_2.pl?file=20181219%2FF002798.dat;pr.eh=12%2C12p;pr.page=12;pr.per_page=1;pr.show=proteins#tc:rf) | 219 | 38837 | 7 (5) | 0.39 | Heterogeneous nuclear ribonucleoprotein A1 OS=Homo sapiens OX=9606 GN=HNRNPA1 PE=1 SV=5 | |
| P07900 | 199 | 85006 | 12 (8) | 0.25 | Heat shock protein HSP 90-alpha OS=Homo sapiens OX=9606 GN=HSP90AA1 PE=1 SV=5 | |
| [P60709](http://sale-depta-pc/mascot/cgi/master_results_2.pl?file=20181219%2FF002798.dat;pr.eh=13%2C13p;pr.page=13;pr.per_page=1;pr.show=proteins#tc:rf) | 196 | 42052 | 13 (11) | 0.7 | Actin, cytoplasmic 1 OS=Homo sapiens OX=9606 GN=ACTB PE=1 SV=1 | |
| [Q86V81](http://sale-depta-pc/mascot/cgi/master_results_2.pl?file=20181219%2FF002798.dat;pr.eh=14%2C14p;pr.page=14;pr.per_page=1;pr.show=proteins#tc:rf) | 184 | 26872 | 3 (3) | 0.26 | THO complex subunit 4 OS=Homo sapiens OX=9606 GN=ALYREF PE=1 SV=3 | |
| [P67809](http://sale-depta-pc/mascot/cgi/master_results_2.pl?file=20181219%2FF002798.dat;pr.eh=15%2C15p;pr.page=15;pr.per_page=1;pr.show=proteins#tc:rf) | 181 | 35903 | 7 (3) | 0.19 | Nuclease-sensitive element-binding protein 1 OS=Homo sapiens OX=9606 GN=YBX1 PE=1 SV=3 | |
| [P06733](http://sale-depta-pc/mascot/cgi/master_results_2.pl?file=20181219%2FF002798.dat;pr.eh=16%2C16p;pr.page=16;pr.per_page=1;pr.show=proteins#tc:rf) | 177 | 47481 | 9 (5) | 0.31 | Alpha-enolase OS=Homo sapiens OX=9606 GN=ENO1 PE=1 SV=2 | |
| [Q9BY77](http://sale-depta-pc/mascot/cgi/master_results_2.pl?file=20181219%2FF002798.dat;pr.eh=17%2C17p;pr.page=17;pr.per_page=1;pr.show=proteins#tc:rf) | 174 | 46289 | 9 (5) | 0.32 | Polymerase delta-interacting protein 3 OS=Homo sapiens OX=9606 GN=POLDIP3 PE=1 SV=2 | |
| [P04792](http://sale-depta-pc/mascot/cgi/master_results_2.pl?file=20181219%2FF002798.dat;pr.eh=18%2C18p;pr.page=18;pr.per_page=1;pr.show=proteins#tc:rf) | 173 | 22826 | 5 (5) | 0.73 | Heat shock protein beta-1 OS=Homo sapiens OX=9606 GN=HSPB1 PE=1 SV=2 | |
| [P17844](http://sale-depta-pc/mascot/cgi/master_results_2.pl?file=20181219%2FF002798.dat;pr.eh=19%2C19p;pr.page=19;pr.per_page=1;pr.show=proteins#tc:rf) | 149 | 69618 | 8 (5) | 0.2 | Probable ATP-dependent RNA helicase DDX5 OS=Homo sapiens OX=9606 GN=DDX5 PE=1 SV=1 | |
| [P14625](http://sale-depta-pc/mascot/cgi/master_results_2.pl?file=20181219%2FF002798.dat;pr.eh=8%2C8p;pr.page=8;pr.per_page=1;pr.show=proteins#tc:rf) | 139 | 92696 | 7 (5) | 0.19 | Endoplasmin OS=Homo sapiens OX=9606 GN=HSP90B1 PE=1 SV=1 | |
| [Q14331](http://sale-depta-pc/mascot/cgi/master_results_2.pl?file=20181219%2FF002798.dat;pr.eh=20%2C20p;pr.page=20;pr.per_page=1;pr.show=proteins#tc:rf) | 128 | 29439 | 3 (3) | 0.24 | Protein FRG1 OS=Homo sapiens OX=9606 GN=FRG1 PE=1 SV=1 | |
| [P55795](http://sale-depta-pc/mascot/cgi/master_results_2.pl?file=20181219%2FF002798.dat;pr.eh=10%2C10p;pr.page=10;pr.per_page=1;pr.show=proteins#tc:rf) | 127 | 49517 | 7 (3) | 0.21 | Heterogeneous nuclear ribonucleoprotein H2 OS=Homo sapiens OX=9606 GN=HNRNPH2 PE=1 SV=1 | |
| P0DMV8 | 121 | 70294 | 6 (5) | 0.2 | Heat shock 70 kDa protein 1A OS=Homo sapiens OX=9606 GN=HSPA1A PE=1 SV=1 | |
| [Q00839](http://sale-depta-pc/mascot/cgi/master_results_2.pl?file=20181219%2FF002798.dat;pr.eh=21%2C21p;pr.page=21;pr.per_page=1;pr.show=proteins#tc:rf) | 118 | 91269 | 5 (3) | 0.07 | Heterogeneous nuclear ribonucleoprotein U OS=Homo sapiens OX=9606 GN=HNRNPU PE=1 SV=6 | |
| [Q9BQE3](http://sale-depta-pc/mascot/cgi/master_results_2.pl?file=20181219%2FF002796.dat;pr.eh=4%2C4p;pr.page=4;pr.per_page=1;pr.show=proteins#tc:rf) | 112 | 50548 | 11 (7) | 0.29 | Tubulin alpha-1C chain OS=Homo sapiens OX=9606 GN=TUBA1C PE=1 SV=1 | |
| [P25705](http://sale-depta-pc/mascot/cgi/master_results_2.pl?file=20181219%2FF002798.dat;pr.eh=22%2C22p;pr.page=22;pr.per_page=1;pr.show=proteins#tc:rf) | 105 | 59828 | 5 (3) | 0.11 | ATP synthase subunit alpha, mitochondrial OS=Homo sapiens OX=9606 GN=ATP5F1A PE=1 SV=1 | |
| [P02768](http://sale-depta-pc/mascot/cgi/master_results_2.pl?file=20181219%2FF002798.dat;pr.eh=23%2C23p;pr.page=23;pr.per_page=1;pr.show=proteins#tc:rf) | 104 | 71317 | 7 (5) | 0.14 | Serum albumin OS=Homo sapiens OX=9606 GN=ALB PE=1 SV=2 | |
| [P62318](http://sale-depta-pc/mascot/cgi/master_results_2.pl?file=20181219%2FF002798.dat;pr.eh=24%2C24p;pr.page=24;pr.per_page=1;pr.show=proteins#tc:rf) | 103 | 14021 | 3 (2) | 0.24 | Small nuclear ribonucleoprotein Sm D3 OS=Homo sapiens OX=9606 GN=SNRPD3 PE=1 SV=1 | |
| [P78406](http://sale-depta-pc/mascot/cgi/master_results_2.pl?file=20181219%2FF002798.dat;pr.eh=25%2C25p;pr.page=25;pr.per_page=1;pr.show=proteins#tc:rf) | 100 | 41569 | 5 (2) | 0.17 | mRNA export factor OS=Homo sapiens OX=9606 GN=RAE1 PE=1 SV=1 | |
| [P38159](http://sale-depta-pc/mascot/cgi/master_results_2.pl?file=20181219%2FF002798.dat;pr.eh=26%2C26p;pr.page=26;pr.per_page=1;pr.show=proteins#tc:rf) | 97 | 42306 | 9 (5) | 0.25 | RNA-binding motif protein, X chromosome OS=Homo sapiens OX=9606 GN=RBMX PE=1 SV=3 | |
| [Q5VTE0](http://sale-depta-pc/mascot/cgi/master_results_2.pl?file=20181219%2FF002798.dat;pr.eh=27%2C27p;pr.page=27;pr.per_page=1;pr.show=proteins#tc:rf) | 97 | 50495 | 3 (3) | 0.13 | Putative elongation factor 1-alpha-like 3 OS=Homo sapiens OX=9606 GN=EEF1A1P5 PE=5 SV=1 | |
| P11021 | 96 | 72402 | 4 (3) | 0.14 | Endoplasmic reticulum chaperone BiP OS=Homo sapiens OX=9606 GN=HSPA5 PE=1 SV=2 | |
| [Q14677](http://sale-depta-pc/mascot/cgi/master_results_2.pl?file=20181219%2FF002798.dat;pr.eh=28%2C28p;pr.page=28;pr.per_page=1;pr.show=proteins#tc:rf) | 93 | 68273 | 5 (4) | 0.15 | Clathrin interactor 1 OS=Homo sapiens OX=9606 GN=CLINT1 PE=1 SV=1 | |
| [P10809](http://sale-depta-pc/mascot/cgi/master_results_2.pl?file=20181219%2FF002798.dat;pr.eh=29%2C29p;pr.page=29;pr.per_page=1;pr.show=proteins#tc:rf) | 85 | 61187 | 6 (3) | 0.11 | 60 kDa heat shock protein, mitochondrial OS=Homo sapiens OX=9606 GN=HSPD1 PE=1 SV=2 | |
| [P47813](http://sale-depta-pc/mascot/cgi/master_results_2.pl?file=20181219%2FF002798.dat;pr.eh=30%2C30p;pr.page=30;pr.per_page=1;pr.show=proteins#tc:rf) | 84 | 16564 | 3 (3) | 0.75 | Eukaryotic translation initiation factor 1A, X-chromosomal OS=Homo sapiens OX=9606 GN=EIF1AX PE=1 SV=2 | |
| [P19338](http://sale-depta-pc/mascot/cgi/master_results_2.pl?file=20181219%2FF002798.dat;pr.eh=31%2C31p;pr.page=31;pr.per_page=1;pr.show=proteins#tc:rf) | 80 | 76625 | 4 (3) | 0.13 | Nucleolin OS=Homo sapiens OX=9606 GN=NCL PE=1 SV=3 | |
| [Q8TF72](http://sale-depta-pc/mascot/cgi/master_results_2.pl?file=20181219%2FF002796.dat;pr.eh=7%2C7p;pr.page=7;pr.per_page=1;pr.show=proteins#tc:rf) | 77 | 218321 | 12 (7) | 0.01 | Protein Shroom3 OS=Homo sapiens OX=9606 GN=SHROOM3 PE=1 SV=2 | |
| [P52597](http://sale-depta-pc/mascot/cgi/master_results_2.pl?file=20181219%2FF002798.dat;pr.eh=10%2C10p;pr.page=10;pr.per_page=1;pr.show=proteins#tc:rf) | 76 | 45985 | 4 (2) | 0.15 | Heterogeneous nuclear ribonucleoprotein F OS=Homo sapiens OX=9606 GN=HNRNPF PE=1 SV=3 | |
| [Q13573](http://sale-depta-pc/mascot/cgi/master_results_2.pl?file=20181219%2FF002798.dat;pr.eh=32%2C32p;pr.page=32;pr.per_page=1;pr.show=proteins#tc:rf) | 73 | 61514 | 8 (3) | 0.17 | SNW domain-containing protein 1 OS=Homo sapiens OX=9606 GN=SNW1 PE=1 SV=1 | |
| [P62851](http://sale-depta-pc/mascot/cgi/master_results_2.pl?file=20181219%2FF002798.dat;pr.eh=33%2C33p;pr.page=33;pr.per_page=1;pr.show=proteins#tc:rf) | 73 | 13791 | 2 (2) | 0.25 | 40S ribosomal protein S25 OS=Homo sapiens OX=9606 GN=RPS25 PE=1 SV=1 | |
| [P09972](http://sale-depta-pc/mascot/cgi/master_results_2.pl?file=20181219%2FF002798.dat;pr.eh=34%2C34p;pr.page=34;pr.per_page=1;pr.show=proteins#tc:rf) | 69 | 39830 | 1 (1) | 0.08 | Fructose-bisphosphate aldolase C OS=Homo sapiens OX=9606 GN=ALDOC PE=1 SV=2 | |
| [P21796](http://sale-depta-pc/mascot/cgi/master_results_2.pl?file=20181219%2FF002798.dat;pr.eh=35%2C35p;pr.page=35;pr.per_page=1;pr.show=proteins#tc:rf) | 65 | 30868 | 10 (3) | 0.36 | Voltage-dependent anion-selective channel protein 1 OS=Homo sapiens OX=9606 GN=VDAC1 PE=1 SV=2 | |
| [P22626](http://sale-depta-pc/mascot/cgi/master_results_2.pl?file=20181219%2FF002798.dat;pr.eh=36%2C36p;pr.page=36;pr.per_page=1;pr.show=proteins#tc:rf) | 64 | 37464 | 4 (3) | 0.29 | Heterogeneous nuclear ribonucleoproteins A2/B1 OS=Homo sapiens OX=9606 GN=HNRNPA2B1 PE=1 SV=2 | |
| [Q96AG4](http://sale-depta-pc/mascot/cgi/master_results_2.pl?file=20181219%2FF002798.dat;pr.eh=37%2C37p;pr.page=37;pr.per_page=1;pr.show=proteins#tc:rf) | 64 | 35308 | 3 (3) | 0.31 | Leucine-rich repeat-containing protein 59 OS=Homo sapiens OX=9606 GN=LRRC59 PE=1 SV=1 | |
| [P53999](http://sale-depta-pc/mascot/cgi/master_results_2.pl?file=20181219%2FF002798.dat;pr.eh=38%2C38p;pr.page=38;pr.per_page=1;pr.show=proteins#tc:rf) | 64 | 14386 | 4 (3) | 0.89 | Activated RNA polymerase II transcriptional coactivator p15 OS=Homo sapiens OX=9606 GN=SUB1 PE=1 SV=3 | |
| [P07195](http://sale-depta-pc/mascot/cgi/master_results_2.pl?file=20181219%2FF002798.dat;pr.eh=39%2C39p;pr.page=39;pr.per_page=1;pr.show=proteins#tc:rf) | 63 | 36900 | 4 (3) | 0.29 | L-lactate dehydrogenase B chain OS=Homo sapiens OX=9606 GN=LDHB PE=1 SV=2 | |
| [Q06830](http://sale-depta-pc/mascot/cgi/master_results_2.pl?file=20181219%2FF002798.dat;pr.eh=40%2C40p;pr.page=40;pr.per_page=1;pr.show=proteins#tc:rf) | 62 | 22324 | 5 (3) | 0.52 | Peroxiredoxin-1 OS=Homo sapiens OX=9606 GN=PRDX1 PE=1 SV=1 | |
| [P06576](http://sale-depta-pc/mascot/cgi/master_results_2.pl?file=20181219%2FF002798.dat;pr.eh=41%2C41p;pr.page=41;pr.per_page=1;pr.show=proteins#tc:rf) | 60 | 56525 | 5 (2) | 0.12 | ATP synthase subunit beta, mitochondrial OS=Homo sapiens OX=9606 GN=ATP5F1B PE=1 SV=3 | |
| [Q15147](http://sale-depta-pc/mascot/cgi/master_results_2.pl?file=20181219%2FF002798.dat;pr.eh=42%2C42p;pr.page=42;pr.per_page=1;pr.show=proteins#tc:rf) | 60 | 135518 | 4 (3) | 0.07 | 1-phosphatidylinositol 4,5-bisphosphate phosphodiesterase beta-4 OS=Homo sapiens OX=9606 GN=PLCB4 PE=1 SV=3 | |
| [P36542](http://sale-depta-pc/mascot/cgi/master_results_2.pl?file=20181219%2FF002798.dat;pr.eh=43%2C43p;pr.page=43;pr.per_page=1;pr.show=proteins#tc:rf) | 60 | 33032 | 1 (1) | 0.1 | ATP synthase subunit gamma, mitochondrial OS=Homo sapiens OX=9606 GN=ATP5F1C PE=1 SV=1 | |
| [Q15366](http://sale-depta-pc/mascot/cgi/master_results_2.pl?file=20181219%2FF002798.dat;pr.eh=44%2C44p;pr.page=44;pr.per_page=1;pr.show=proteins#tc:rf) | 58 | 38955 | 3 (1) | 0.08 | Poly(rC)-binding protein 2 OS=Homo sapiens OX=9606 GN=PCBP2 PE=1 SV=1 | |
| [P07737](http://sale-depta-pc/mascot/cgi/master_results_2.pl?file=20181219%2FF002798.dat;pr.eh=45%2C45p;pr.page=45;pr.per_page=1;pr.show=proteins#tc:rf) | 57 | 15216 | 2 (2) | 0.5 | Profilin-1 OS=Homo sapiens OX=9606 GN=PFN1 PE=1 SV=2 | |
| [P62263](http://sale-depta-pc/mascot/cgi/master_results_2.pl?file=20181219%2FF002798.dat;pr.eh=46%2C46p;pr.page=46;pr.per_page=1;pr.show=proteins#tc:rf) | 56 | 16434 | 3 (1) | 0.21 | 40S ribosomal protein S14 OS=Homo sapiens OX=9606 GN=RPS14 PE=1 SV=3 | |
| [P62753](http://sale-depta-pc/mascot/cgi/master_results_2.pl?file=20181219%2FF002798.dat;pr.eh=47%2C47p;pr.page=47;pr.per_page=1;pr.show=proteins#tc:rf) | 55 | 28834 | 2 (1) | 0.12 | 40S ribosomal protein S6 OS=Homo sapiens OX=9606 GN=RPS6 PE=1 SV=1 | |
| [P62913](http://sale-depta-pc/mascot/cgi/master_results_2.pl?file=20181219%2FF002798.dat;pr.eh=48%2C48p;pr.page=48;pr.per_page=1;pr.show=proteins#tc:rf) | 52 | 20468 | 1 (1) | 0.16 | 60S ribosomal protein L11 OS=Homo sapiens OX=9606 GN=RPL11 PE=1 SV=2 | |
| [Q9NQ39](http://sale-depta-pc/mascot/cgi/master_results_2.pl?file=20181219%2FF002798.dat;pr.eh=49%2C49p;pr.page=49;pr.per_page=1;pr.show=proteins#tc:rf) | 51 | 20279 | 2 (1) | 0.17 | Putative 40S ribosomal protein S10-like OS=Homo sapiens OX=9606 GN=RPS10P5 PE=5 SV=1 | |
| [Q8ND56](http://sale-depta-pc/mascot/cgi/master_results_2.pl?file=20181219%2FF002798.dat;pr.eh=50%2C50p;pr.page=50;pr.per_page=1;pr.show=proteins#tc:rf) | 51 | 50727 | 3 (2) | 0.13 | Protein LSM14 homolog A OS=Homo sapiens OX=9606 GN=LSM14A PE=1 SV=3 | |
| [Q01518](http://sale-depta-pc/mascot/cgi/master_results_2.pl?file=20181219%2FF002798.dat;pr.eh=51%2C51p;pr.page=51;pr.per_page=1;pr.show=proteins#tc:rf) | 51 | 52325 | 3 (2) | 0.13 | Adenylyl cyclase-associated protein 1 OS=Homo sapiens OX=9606 GN=CAP1 PE=1 SV=5 | |
| [P11498](http://sale-depta-pc/mascot/cgi/master_results_2.pl?file=20181219%2FF002798.dat;pr.eh=52%2C52p;pr.page=52;pr.per_page=1;pr.show=proteins#tc:rf) | 49 | 130293 | 5 (2) | 0.05 | Pyruvate carboxylase, mitochondrial OS=Homo sapiens OX=9606 GN=PC PE=1 SV=2 | |
| [Q96RQ3](http://sale-depta-pc/mascot/cgi/master_results_2.pl?file=20181219%2FF002798.dat;pr.eh=53%2C53p;pr.page=53;pr.per_page=1;pr.show=proteins#tc:rf) | 49 | 80935 | 4 (1) | 0.04 | Methylcrotonoyl-CoA carboxylase subunit alpha, mitochondrial OS=Homo sapiens OX=9606 GN=MCCC1 PE=1 SV=3 | |
| [Q9Y3Y2](http://sale-depta-pc/mascot/cgi/master_results_2.pl?file=20181219%2FF002798.dat;pr.eh=54%2C54p;pr.page=54;pr.per_page=1;pr.show=proteins#tc:rf) | 48 | 26380 | 1 (1) | 0.13 | Chromatin target of PRMT1 protein OS=Homo sapiens OX=9606 GN=CHTOP PE=1 SV=2 | |
| [Q9Y6M1](http://sale-depta-pc/mascot/cgi/master_results_2.pl?file=20181219%2FF002798.dat;pr.eh=55%2C55p;pr.page=55;pr.per_page=1;pr.show=proteins#tc:rf) | 48 | 66195 | 3 (1) | 0.05 | Insulin-like growth factor 2 mRNA-binding protein 2 OS=Homo sapiens OX=9606 GN=IGF2BP2 PE=1 SV=2 | |
| [Q13085](http://sale-depta-pc/mascot/cgi/master_results_2.pl?file=20181219%2FF002798.dat;pr.eh=56%2C56p;pr.page=56;pr.per_page=1;pr.show=proteins#tc:rf) | 47 | 267095 | 9 (2) | 0.02 | Acetyl-CoA carboxylase 1 OS=Homo sapiens OX=9606 GN=ACACA PE=1 SV=2 | |
| [Q13112](http://sale-depta-pc/mascot/cgi/master_results_2.pl?file=20181219%2FF002798.dat;pr.eh=57%2C57p;pr.page=57;pr.per_page=1;pr.show=proteins#tc:rf) | 45 | 61910 | 2 (1) | 0.05 | Chromatin assembly factor 1 subunit B OS=Homo sapiens OX=9606 GN=CHAF1B PE=1 SV=1 | |
| [Q92974](http://sale-depta-pc/mascot/cgi/master_results_2.pl?file=20181219%2FF002798.dat;pr.eh=58%2C58p;pr.page=58;pr.per_page=1;pr.show=proteins#tc:rf) | 45 | 112386 | 3 (1) | 0.03 | Rho guanine nucleotide exchange factor 2 OS=Homo sapiens OX=9606 GN=ARHGEF2 PE=1 SV=4 | |
| [Q9H0A0](http://sale-depta-pc/mascot/cgi/master_results_2.pl?file=20181219%2FF002798.dat;pr.eh=59%2C59p;pr.page=59;pr.per_page=1;pr.show=proteins#tc:rf) | 44 | 116569 | 4 (1) | 0.03 | RNA cytidine acetyltransferase OS=Homo sapiens OX=9606 GN=NAT10 PE=1 SV=2 | |
| [P14618](http://sale-depta-pc/mascot/cgi/master_results_2.pl?file=20181219%2FF002798.dat;pr.eh=60%2C60p;pr.page=60;pr.per_page=1;pr.show=proteins#tc:rf) | 43 | 58470 | 5 (2) | 0.12 | Pyruvate kinase PKM OS=Homo sapiens OX=9606 GN=PKM PE=1 SV=4 | |
| [P61978](http://sale-depta-pc/mascot/cgi/master_results_2.pl?file=20181219%2FF002798.dat;pr.eh=61%2C61p;pr.page=61;pr.per_page=1;pr.show=proteins#tc:rf) | 43 | 51230 | 3 (2) | 0.13 | Heterogeneous nuclear ribonucleoprotein K OS=Homo sapiens OX=9606 GN=HNRNPK PE=1 SV=1 | |
| [P05141](http://sale-depta-pc/mascot/cgi/master_results_2.pl?file=20181219%2FF002798.dat;pr.eh=62%2C62p;pr.page=62;pr.per_page=1;pr.show=proteins#tc:rf) | 43 | 33059 | 3 (1) | 0.1 | ADP/ATP translocase 2 OS=Homo sapiens OX=9606 GN=SLC25A5 PE=1 SV=7 | |
| [P63244](http://sale-depta-pc/mascot/cgi/master_results_2.pl?file=20181219%2FF002798.dat;pr.eh=63%2C63p;pr.page=63;pr.per_page=1;pr.show=proteins#tc:rf) | 42 | 35511 | 4 (2) | 0.2 | Receptor of activated protein C kinase 1 OS=Homo sapiens OX=9606 GN=RACK1 PE=1 SV=3 | |
| [P35268](http://sale-depta-pc/mascot/cgi/master_results_2.pl?file=20181219%2FF002798.dat;pr.eh=64%2C64p;pr.page=64;pr.per_page=1;pr.show=proteins#tc:rf) | 41 | 14835 | 1 (1) | 0.23 | 60S ribosomal protein L22 OS=Homo sapiens OX=9606 GN=RPL22 PE=1 SV=2 | |
| [Q14980](http://sale-depta-pc/mascot/cgi/master_results_2.pl?file=20181219%2FF002798.dat;pr.eh=65%2C65p;pr.page=65;pr.per_page=1;pr.show=proteins#tc:rf) | 40 | 239199 | 11 (1) | 0.01 | Nuclear mitotic apparatus protein 1 OS=Homo sapiens OX=9606 GN=NUMA1 PE=1 SV=2 | |
| [P49790](http://sale-depta-pc/mascot/cgi/master_results_2.pl?file=20181219%2FF002798.dat;pr.eh=66%2C66p;pr.page=66;pr.per_page=1;pr.show=proteins#tc:rf) | 40 | 155440 | 3 (1) | 0.02 | Nuclear pore complex protein Nup153 OS=Homo sapiens OX=9606 GN=NUP153 PE=1 SV=2 | |
| [P83731](http://sale-depta-pc/mascot/cgi/master_results_2.pl?file=20181219%2FF002798.dat;pr.eh=67%2C67p;pr.page=67;pr.per_page=1;pr.show=proteins#tc:rf) | 39 | 17882 | 2 (1) | 0.19 | 60S ribosomal protein L24 OS=Homo sapiens OX=9606 GN=RPL24 PE=1 SV=1 | |
| [Q9HCC0](http://sale-depta-pc/mascot/cgi/master_results_2.pl?file=20181219%2FF002798.dat;pr.eh=69%2C69p;pr.page=69;pr.per_page=1;pr.show=proteins#tc:rf) | 36 | 61808 | 4 (1) | 0.05 | Methylcrotonoyl-CoA carboxylase beta chain, mitochondrial OS=Homo sapiens OX=9606 GN=MCCC2 PE=1 SV=1 | |
| [Q14103](http://sale-depta-pc/mascot/cgi/master_results_2.pl?file=20181219%2FF002798.dat;pr.eh=70%2C70p;pr.page=70;pr.per_page=1;pr.show=proteins#tc:rf) | 36 | 38581 | 1 (1) | 0.09 | Heterogeneous nuclear ribonucleoprotein D0 OS=Homo sapiens OX=9606 GN=HNRNPD PE=1 SV=1 | |
| [O43175](http://sale-depta-pc/mascot/cgi/master_results_2.pl?file=20181219%2FF002798.dat;pr.eh=71%2C71p;pr.page=71;pr.per_page=1;pr.show=proteins#tc:rf) | 35 | 57356 | 3 (1) | 0.06 | D-3-phosphoglycerate dehydrogenase OS=Homo sapiens OX=9606 GN=PHGDH PE=1 SV=4 | |
| [O43290](http://sale-depta-pc/mascot/cgi/master_results_2.pl?file=20181219%2FF002798.dat;pr.eh=72%2C72p;pr.page=72;pr.per_page=1;pr.show=proteins#tc:rf) | 35 | 90371 | 1 (1) | 0.04 | U4/U6.U5 tri-snRNP-associated protein 1 OS=Homo sapiens OX=9606 GN=SART1 PE=1 SV=1 | |
| [Q8NC51](http://sale-depta-pc/mascot/cgi/master_results_2.pl?file=20181219%2FF002798.dat;pr.eh=73%2C73p;pr.page=73;pr.per_page=1;pr.show=proteins#tc:rf) | 35 | 44995 | 3 (1) | 0.07 | Plasminogen activator inhibitor 1 RNA-binding protein OS=Homo sapiens OX=9606 GN=SERBP1 PE=1 SV=2 | |
| [P54652](http://sale-depta-pc/mascot/cgi/master_results_2.pl?file=20181219%2FF002796.dat;pr.eh=14%2C14p;pr.page=14;pr.per_page=1;pr.show=proteins#tc:rf) | 34 | 70263 | 5 (1) | 0.05 | Heat shock-related 70 kDa protein 2 OS=Homo sapiens OX=9606 GN=HSPA2 PE=1 SV=1 | |
| [O43809](http://sale-depta-pc/mascot/cgi/master_results_2.pl?file=20181219%2FF002798.dat;pr.eh=74%2C74p;pr.page=74;pr.per_page=1;pr.show=proteins#tc:rf) | 34 | 26268 | 2 (1) | 0.13 | Cleavage and polyadenylation specificity factor subunit 5 OS=Homo sapiens OX=9606 GN=NUDT21 PE=1 SV=1 | |
| [P16402](http://sale-depta-pc/mascot/cgi/master_results_2.pl?file=20181219%2FF002798.dat;pr.eh=75%2C75p;pr.page=75;pr.per_page=1;pr.show=proteins#tc:rf) | 34 | 22336 | 33 (1) | 0.15 | Histone H1.3 OS=Homo sapiens OX=9606 GN=HIST1H1D PE=1 SV=2 | |
| [Q9Y536](http://sale-depta-pc/mascot/cgi/master_results_2.pl?file=20181219%2FF002798.dat;pr.eh=76%2C76p;pr.page=76;pr.per_page=1;pr.show=proteins#tc:rf) | 34 | 18398 | 1 (1) | 0.18 | Peptidyl-prolyl cis-trans isomerase A-like 4A OS=Homo sapiens OX=9606 GN=PPIAL4A PE=2 SV=1 | |
| [P42167](http://sale-depta-pc/mascot/cgi/master_results_2.pl?file=20181219%2FF002798.dat;pr.eh=77%2C77p;pr.page=77;pr.per_page=1;pr.show=proteins#tc:rf) | 34 | 50696 | 2 (1) | 0.06 | Lamina-associated polypeptide 2, isoforms beta/gamma OS=Homo sapiens OX=9606 GN=TMPO PE=1 SV=2 | |
| [P63104](http://sale-depta-pc/mascot/cgi/master_results_2.pl?file=20181219%2FF002798.dat;pr.eh=78%2C78p;pr.page=78;pr.per_page=1;pr.show=proteins#tc:rf) | 33 | 27899 | 3 (2) | 0.12 | 14-3-3 protein zeta/delta OS=Homo sapiens OX=9606 GN=YWHAZ PE=1 SV=1 | |
| [Q14011](http://sale-depta-pc/mascot/cgi/master_results_2.pl?file=20181219%2FF002798.dat;pr.eh=79%2C79p;pr.page=79;pr.per_page=1;pr.show=proteins#tc:rf) | 32 | 18637 | 2 (1) | 0.18 | Cold-inducible RNA-binding protein OS=Homo sapiens OX=9606 GN=CIRBP PE=1 SV=1 | |
| [P09669](http://sale-depta-pc/mascot/cgi/master_results_2.pl?file=20181219%2FF002798.dat;pr.eh=80%2C80p;pr.page=80;pr.per_page=1;pr.show=proteins#tc:rf) | 32 | 8776 | 1 (1) | 0.4 | Cytochrome c oxidase subunit 6C OS=Homo sapiens OX=9606 GN=COX6C PE=1 SV=2 | |
| [P62979](http://sale-depta-pc/mascot/cgi/master_results_2.pl?file=20181219%2FF002798.dat;pr.eh=81%2C81p;pr.page=81;pr.per_page=1;pr.show=proteins#tc:rf) | 31 | 18296 | 4 (2) | 0.4 | Ubiquitin-40S ribosomal protein S27a OS=Homo sapiens OX=9606 GN=RPS27A PE=1 SV=2 | |
| [Q8WWY3](http://sale-depta-pc/mascot/cgi/master_results_2.pl?file=20181219%2FF002798.dat;pr.eh=82%2C82p;pr.page=82;pr.per_page=1;pr.show=proteins#tc:rf) | 31 | 55649 | 1 (1) | 0.06 | U4/U6 small nuclear ribonucleoprotein Prp31 OS=Homo sapiens OX=9606 GN=PRPF31 PE=1 SV=2 | |
| [Q53F19](http://sale-depta-pc/mascot/cgi/master_results_2.pl?file=20181219%2FF002798.dat;pr.eh=83%2C83p;pr.page=83;pr.per_page=1;pr.show=proteins#tc:rf) | 31 | 70663 | 3 (1) | 0.05 | Nuclear cap-binding protein subunit 3 OS=Homo sapiens OX=9606 GN=NCBP3 PE=1 SV=2 | |
| [P61204](http://sale-depta-pc/mascot/cgi/master_results_2.pl?file=20181219%2FF002798.dat;pr.eh=84%2C84p;pr.page=84;pr.per_page=1;pr.show=proteins#tc:rf) | 31 | 20645 | 3 (1) | 0.16 | ADP-ribosylation factor 3 OS=Homo sapiens OX=9606 GN=ARF3 PE=1 SV=2 | |
| [Q7Z7H8](http://sale-depta-pc/mascot/cgi/master_results_2.pl?file=20181219%2FF002798.dat;pr.eh=85%2C85p;pr.page=85;pr.per_page=1;pr.show=proteins#tc:rf) | 31 | 29492 | 2 (1) | 0.11 | 39S ribosomal protein L10, mitochondrial OS=Homo sapiens OX=9606 GN=MRPL10 PE=1 SV=3 | |
| [P0DN76](http://sale-depta-pc/mascot/cgi/master_results_2.pl?file=20181219%2FF002796.dat;pr.eh=16%2C16p;pr.page=16;pr.per_page=1;pr.show=proteins#tc:rf) | 30 | 28368 | 2 (2) | 0.12 | Splicing factor U2AF 35 kDa subunit-like protein OS=Homo sapiens OX=9606 GN=U2AF1L5 PE=1 SV=1 | |
| [Q13268](http://sale-depta-pc/mascot/cgi/master_results_2.pl?file=20181219%2FF002798.dat;pr.eh=86%2C86p;pr.page=86;pr.per_page=1;pr.show=proteins#tc:rf) | 30 | 30307 | 2 (1) | 0.11 | Dehydrogenase/reductase SDR family member 2, mitochondrial OS=Homo sapiens OX=9606 GN=DHRS2 PE=1 SV=4 | |
| [Q96DF8](http://sale-depta-pc/mascot/cgi/master_results_2.pl?file=20181219%2FF002798.dat;pr.eh=87%2C87p;pr.page=87;pr.per_page=1;pr.show=proteins#tc:rf) | 30 | 52593 | 4 (1) | 0.06 | Splicing factor ESS-2 homolog OS=Homo sapiens OX=9606 GN=ESS2 PE=1 SV=1 | |
| [P27797](http://sale-depta-pc/mascot/cgi/master_results_2.pl?file=20181219%2FF002798.dat;pr.eh=88%2C88p;pr.page=88;pr.per_page=1;pr.show=proteins#tc:rf) | 30 | 48283 | 1 (1) | 0.07 | Calreticulin OS=Homo sapiens OX=9606 GN=CALR PE=1 SV=1 | |
| [Q9BVA1](http://sale-depta-pc/mascot/cgi/master_results_2.pl?file=20181219%2FF002796.dat;pr.eh=18%2C18p;pr.page=18;pr.per_page=1;pr.show=proteins#tc:rf) | 28 | 50377 | 2 (1) | 0.07 | Tubulin beta-2B chain OS=Homo sapiens OX=9606 GN=TUBB2B PE=1 SV=1 | |
| [Q71UM5](http://sale-depta-pc/mascot/cgi/master_results_2.pl?file=20181219%2FF002798.dat;pr.eh=89%2C89p;pr.page=89;pr.per_page=1;pr.show=proteins#tc:rf) | 28 | 9813 | 1 (1) | 0.36 | 40S ribosomal protein S27-like OS=Homo sapiens OX=9606 GN=RPS27L PE=1 SV=3 | |
| [P30050](http://sale-depta-pc/mascot/cgi/master_results_2.pl?file=20181219%2FF002798.dat;pr.eh=90%2C90p;pr.page=90;pr.per_page=1;pr.show=proteins#tc:rf) | 27 | 17979 | 1 (1) | 0.19 | 60S ribosomal protein L12 OS=Homo sapiens OX=9606 GN=RPL12 PE=1 SV=1 | |
| [P84103](http://sale-depta-pc/mascot/cgi/master_results_2.pl?file=20181219%2FF002798.dat;pr.eh=92%2C92p;pr.page=92;pr.per_page=1;pr.show=proteins#tc:rf) | 27 | 19546 | 2 (1) | 0.17 | Serine/arginine-rich splicing factor 3 OS=Homo sapiens OX=9606 GN=SRSF3 PE=1 SV=1 | |
| [Q09028](http://sale-depta-pc/mascot/cgi/master_results_2.pl?file=20181219%2FF002798.dat;pr.eh=93%2C93p;pr.page=93;pr.per_page=1;pr.show=proteins#tc:rf) | 27 | 47911 | 1 (1) | 0.07 | Histone-binding protein RBBP4 OS=Homo sapiens OX=9606 GN=RBBP4 PE=1 SV=3 | |
| [P0DPB5](http://sale-depta-pc/mascot/cgi/master_results_2.pl?file=20181219%2FF002798.dat;pr.eh=94%2C94p;pr.page=94;pr.per_page=1;pr.show=proteins#tc:rf) | 27 | 14380 | 2 (1) | 0.24 | Protein POLR1D, isoform 2 OS=Homo sapiens OX=9606 GN=POLR1D PE=1 SV=1 | |
| [Q9UJ96](http://sale-depta-pc/mascot/cgi/master_results_2.pl?file=20181219%2FF002796.dat;pr.eh=20%2C20p;pr.page=20;pr.per_page=1;pr.show=proteins#tc:rf) | 26 | 52176 | 7 (3) | 0.06 | Potassium voltage-gated channel subfamily G member 2 OS=Homo sapiens OX=9606 GN=KCNG2 PE=1 SV=1 | |
| [Q14011](http://sale-depta-pc/mascot/cgi/master_results_2.pl?file=20181219%2FF002796.dat;pr.eh=21%2C21p;pr.page=21;pr.per_page=1;pr.show=proteins#tc:rf) | 26 | 18637 | 2 (1) | 0.18 | Cold-inducible RNA-binding protein OS=Homo sapiens OX=9606 GN=CIRBP PE=1 SV=1 | |
| [Q6DN72](http://sale-depta-pc/mascot/cgi/master_results_2.pl?file=20181219%2FF002798.dat;pr.eh=95%2C95p;pr.page=95;pr.per_page=1;pr.show=proteins#tc:rf) | 26 | 48515 | 4 (1) | 0.07 | Fc receptor-like protein 6 OS=Homo sapiens OX=9606 GN=FCRL6 PE=1 SV=2 | |
| [Q13151](http://sale-depta-pc/mascot/cgi/master_results_2.pl?file=20181219%2FF002798.dat;pr.eh=96%2C96p;pr.page=96;pr.per_page=1;pr.show=proteins#tc:rf) | 26 | 30993 | 1 (1) | 0.11 | Heterogeneous nuclear ribonucleoprotein A0 OS=Homo sapiens OX=9606 GN=HNRNPA0 PE=1 SV=1 | |
| [Q14498](http://sale-depta-pc/mascot/cgi/master_results_2.pl?file=20181219%2FF002798.dat;pr.eh=97%2C97p;pr.page=97;pr.per_page=1;pr.show=proteins#tc:rf) | 26 | 59628 | 1 (1) | 0.06 | RNA-binding protein 39 OS=Homo sapiens OX=9606 GN=RBM39 PE=1 SV=2 | |
| [Q14134](http://sale-depta-pc/mascot/cgi/master_results_2.pl?file=20181219%2FF002798.dat;pr.eh=98%2C98p;pr.page=98;pr.per_page=1;pr.show=proteins#tc:rf) | 26 | 66478 | 2 (1) | 0.05 | Tripartite motif-containing protein 29 OS=Homo sapiens OX=9606 GN=TRIM29 PE=1 SV=2 | |
| [Q6ZS27](http://sale-depta-pc/mascot/cgi/master_results_2.pl?file=20181219%2FF002796.dat;pr.eh=22%2C22p;pr.page=22;pr.per_page=1;pr.show=proteins#tc:rf) | 25 | 49833 | 1 (1) | 0.07 | Zinc finger protein 662 OS=Homo sapiens OX=9606 GN=ZNF662 PE=1 SV=1 | |
| [Q5VZ89](http://sale-depta-pc/mascot/cgi/master_results_2.pl?file=20181219%2FF002798.dat;pr.eh=99%2C99p;pr.page=99;pr.per_page=1;pr.show=proteins#tc:rf) | 25 | 214914 | 6 (1) | 0.02 | DENN domain-containing protein 4C OS=Homo sapiens OX=9606 GN=DENND4C PE=1 SV=3 | |
| [P38646](http://sale-depta-pc/mascot/cgi/master_results_2.pl?file=20181219%2FF002798.dat;pr.eh=100%2C100p;pr.page=100;pr.per_page=1;pr.show=proteins#tc:rf) | 25 | 73920 | 2 (1) | 0.04 | Stress-70 protein, mitochondrial OS=Homo sapiens OX=9606 GN=HSPA9 PE=1 SV=2 | |
| [Q9Y4F4](http://sale-depta-pc/mascot/cgi/master_results_2.pl?file=20181219%2FF002796.dat;pr.eh=23%2C23p;pr.page=23;pr.per_page=1;pr.show=proteins#tc:rf) | 24 | 190611 | 38 (1) | 0.02 | TOG array regulator of axonemal microtubules protein 1 OS=Homo sapiens OX=9606 GN=TOGARAM1 PE=1 SV=4 | |
| [Q96QU1](http://sale-depta-pc/mascot/cgi/master_results_2.pl?file=20181219%2FF002796.dat;pr.eh=24%2C24p;pr.page=24;pr.per_page=1;pr.show=proteins#tc:rf) | 24 | 217303 | 34 (1) | 0.02 | Protocadherin-15 OS=Homo sapiens OX=9606 GN=PCDH15 PE=1 SV=2 | |
| [Q15004](http://sale-depta-pc/mascot/cgi/master_results_2.pl?file=20181219%2FF002798.dat;pr.eh=101%2C101p;pr.page=101;pr.per_page=1;pr.show=proteins#tc:rf) | 24 | 12093 | 3 (1) | 0.28 | PCNA-associated factor OS=Homo sapiens OX=9606 GN=PCLAF PE=1 SV=1 | |
| [P04075](http://sale-depta-pc/mascot/cgi/master_results_2.pl?file=20181219%2FF002798.dat;pr.eh=102%2C102p;pr.page=102;pr.per_page=1;pr.show=proteins#tc:rf) | 24 | 39851 | 3 (1) | 0.08 | Fructose-bisphosphate aldolase A OS=Homo sapiens OX=9606 GN=ALDOA PE=1 SV=2 | |
| [P0CH98](http://sale-depta-pc/mascot/cgi/master_results_2.pl?file=20181219%2FF002798.dat;pr.eh=103%2C103p;pr.page=103;pr.per_page=1;pr.show=proteins#tc:rf) | 24 | 19233 | 1 (1) | 0.18 | Putative protein FAM106C OS=Homo sapiens OX=9606 GN=FAM106CP PE=5 SV=1 | |
| [Q9NP73](http://sale-depta-pc/mascot/cgi/master_results_2.pl?file=20181219%2FF002798.dat;pr.eh=104%2C104p;pr.page=104;pr.per_page=1;pr.show=proteins#tc:rf) | 24 | 127743 | 4 (1) | 0.03 | Putative bifunctional UDP-N-acetylglucosamine transferase and deubiquitinase ALG13 OS=Homo sapiens OX=9606 GN=ALG13 PE=1 SV=2 | |
| [Q8IZD9](http://sale-depta-pc/mascot/cgi/master_results_2.pl?file=20181219%2FF002798.dat;pr.eh=105%2C105p;pr.page=105;pr.per_page=1;pr.show=proteins#tc:rf) | 24 | 235006 | 4 (1) | 0.01 | Dedicator of cytokinesis protein 3 OS=Homo sapiens OX=9606 GN=DOCK3 PE=1 SV=1 | |
| [Q8IYS1](http://sale-depta-pc/mascot/cgi/master_results_2.pl?file=20181219%2FF002796.dat;pr.eh=25%2C25p;pr.page=25;pr.per_page=1;pr.show=proteins#tc:rf) | 23 | 48088 | 19 (1) | 0.07 | Peptidase M20 domain-containing protein 2 OS=Homo sapiens OX=9606 GN=PM20D2 PE=1 SV=2 | |
| [Q9UIF3](http://sale-depta-pc/mascot/cgi/master_results_2.pl?file=20181219%2FF002796.dat;pr.eh=26%2C26p;pr.page=26;pr.per_page=1;pr.show=proteins#tc:rf) | 23 | 50155 | 14 (1) | 0.07 | Tektin-2 OS=Homo sapiens OX=9606 GN=TEKT2 PE=1 SV=1 | |
| [O43172](http://sale-depta-pc/mascot/cgi/master_results_2.pl?file=20181219%2FF002796.dat;pr.eh=27%2C27p;pr.page=27;pr.per_page=1;pr.show=proteins#tc:rf) | 23 | 59097 | 1 (1) | 0.06 | U4/U6 small nuclear ribonucleoprotein Prp4 OS=Homo sapiens OX=9606 GN=PRPF4 PE=1 SV=2 | |
| [Q9H2U1](http://sale-depta-pc/mascot/cgi/master_results_2.pl?file=20181219%2FF002796.dat;pr.eh=28%2C28p;pr.page=28;pr.per_page=1;pr.show=proteins#tc:rf) | 23 | 115600 | 19 (1) | 0.03 | ATP-dependent RNA helicase DHX36 OS=Homo sapiens OX=9606 GN=DHX36 PE=1 SV=2 | |
| [Q04721](http://sale-depta-pc/mascot/cgi/master_results_2.pl?file=20181219%2FF002798.dat;pr.eh=106%2C106p;pr.page=106;pr.per_page=1;pr.show=proteins#tc:rf) | 23 | 279082 | 115 (1) | 0.01 | Neurogenic locus notch homolog protein 2 OS=Homo sapiens OX=9606 GN=NOTCH2 PE=1 SV=3 | |
| [O95071](http://sale-depta-pc/mascot/cgi/master_results_2.pl?file=20181219%2FF002798.dat;pr.eh=107%2C107p;pr.page=107;pr.per_page=1;pr.show=proteins#tc:rf) | 23 | 312352 | 4 (1) | 0.01 | E3 ubiquitin-protein ligase UBR5 OS=Homo sapiens OX=9606 GN=UBR5 PE=1 SV=2 | |
| [Q9NYJ8](http://sale-depta-pc/mascot/cgi/master_results_2.pl?file=20181219%2FF002798.dat;pr.eh=108%2C108p;pr.page=108;pr.per_page=1;pr.show=proteins#tc:rf) | 23 | 77017 | 11 (1) | 0.04 | TGF-beta-activated kinase 1 and MAP3K7-binding protein 2 OS=Homo sapiens OX=9606 GN=TAB2 PE=1 SV=1 | |
| [Q02413](http://sale-depta-pc/mascot/cgi/master_results_2.pl?file=20181219%2FF002798.dat;pr.eh=109%2C109p;pr.page=109;pr.per_page=1;pr.show=proteins#tc:rf) | 23 | 114702 | 3 (1) | 0.03 | Desmoglein-1 OS=Homo sapiens OX=9606 GN=DSG1 PE=1 SV=2 | |
| [Q9BYG4](http://sale-depta-pc/mascot/cgi/master_results_2.pl?file=20181219%2FF002798.dat;pr.eh=110%2C110p;pr.page=110;pr.per_page=1;pr.show=proteins#tc:rf) | 23 | 41086 | 1 (1) | 0.08 | Partitioning defective 6 homolog gamma OS=Homo sapiens OX=9606 GN=PARD6G PE=1 SV=1 | |
| [P23284](http://sale-depta-pc/mascot/cgi/master_results_2.pl?file=20181219%2FF002798.dat;pr.eh=111%2C111p;pr.page=111;pr.per_page=1;pr.show=proteins#tc:rf) | 23 | 23785 | 2 (1) | 0.14 | Peptidyl-prolyl cis-trans isomerase B OS=Homo sapiens OX=9606 GN=PPIB PE=1 SV=2 | |
| [Q5JST6](http://sale-depta-pc/mascot/cgi/master_results_2.pl?file=20181219%2FF002796.dat;pr.eh=29%2C29p;pr.page=29;pr.per_page=1;pr.show=proteins#tc:rf) | 22 | 88253 | 2 (1) | 0.04 | EF-hand domain-containing family member C2 OS=Homo sapiens OX=9606 GN=EFHC2 PE=1 SV=2 | |
| [Q702N8](http://sale-depta-pc/mascot/cgi/master_results_2.pl?file=20181219%2FF002796.dat;pr.eh=30%2C30p;pr.page=30;pr.per_page=1;pr.show=proteins#tc:rf) | 22 | 199636 | 3 (1) | 0.02 | Xin actin-binding repeat-containing protein 1 OS=Homo sapiens OX=9606 GN=XIRP1 PE=1 SV=1 | |
| [Q15154](http://sale-depta-pc/mascot/cgi/master_results_2.pl?file=20181219%2FF002796.dat;pr.eh=31%2C31p;pr.page=31;pr.per_page=1;pr.show=proteins#tc:rf) | 22 | 230057 | 37 (1) | 0.01 | Pericentriolar material 1 protein OS=Homo sapiens OX=9606 GN=PCM1 PE=1 SV=5 | |
| [Q8ND04](http://sale-depta-pc/mascot/cgi/master_results_2.pl?file=20181219%2FF002796.dat;pr.eh=32%2C32p;pr.page=32;pr.per_page=1;pr.show=proteins#tc:rf) | 22 | 110983 | 9 (1) | 0.03 | Protein SMG8 OS=Homo sapiens OX=9606 GN=SMG8 PE=1 SV=1 | |
| [P0C0S8](http://sale-depta-pc/mascot/cgi/master_results_2.pl?file=20181219%2FF002798.dat;pr.eh=112%2C112p;pr.page=112;pr.per_page=1;pr.show=proteins#tc:rf) | 21 | 14083 | 2 (1) | 0.24 | Histone H2A type 1 OS=Homo sapiens OX=9606 GN=HIST1H2AG PE=1 SV=2 | |
| [P29401](http://sale-depta-pc/mascot/cgi/master_results_2.pl?file=20181219%2FF002798.dat;pr.eh=113%2C113p;pr.page=113;pr.per_page=1;pr.show=proteins#tc:rf) | 21 | 68519 | 1 (1) | 0.05 | Transketolase OS=Homo sapiens OX=9606 GN=TKT PE=1 SV=3 | |
| [Q5VST9](http://sale-depta-pc/mascot/cgi/master_results_2.pl?file=20181219%2FF002798.dat;pr.eh=114%2C114p;pr.page=114;pr.per_page=1;pr.show=proteins#tc:rf) | 20 | 879630 | 9 (1) | 0.02 | Obscurin OS=Homo sapiens OX=9606 GN=OBSCN PE=1 SV=3 | |
| [P21439](http://sale-depta-pc/mascot/cgi/master_results_2.pl?file=20181219%2FF002798.dat;pr.eh=115%2C115p;pr.page=115;pr.per_page=1;pr.show=proteins#tc:rf) | 19 | 142004 | 10 (1) | 0.02 | Phosphatidylcholine translocator ABCB4 OS=Homo sapiens OX=9606 GN=ABCB4 PE=1 SV=2 | |
| [P11532](http://sale-depta-pc/mascot/cgi/master_results_2.pl?file=20181219%2FF002798.dat;pr.eh=116%2C116p;pr.page=116;pr.per_page=1;pr.show=proteins#tc:rf) | 18 | 428537 | 22 (2) | 0.01 | Dystrophin OS=Homo sapiens OX=9606 GN=DMD PE=1 SV=3 | |
| [Q4G0P3](http://sale-depta-pc/mascot/cgi/master_results_2.pl?file=20181219%2FF002796.dat;pr.eh=33%2C33p;pr.page=33;pr.per_page=1;pr.show=proteins#tc:rf) | 17 | 580945 | 2 (1) | 0.01 | Hydrocephalus-inducing protein homolog OS=Homo sapiens OX=9606 GN=HYDIN PE=1 SV=3 | |
| [P42345](http://sale-depta-pc/mascot/cgi/master_results_2.pl?file=20181219%2FF002796.dat;pr.eh=34%2C34p;pr.page=34;pr.per_page=1;pr.show=proteins#tc:rf) | 16 | 290759 | 6 (1) | 0.01 | Serine/threonine-protein kinase mTOR OS=Homo sapiens OX=9606 GN=MTOR PE=1 SV=1 | |
| [Q9NPJ1](http://sale-depta-pc/mascot/cgi/master_results_2.pl?file=20181219%2FF002798.dat;pr.eh=117%2C117p;pr.page=117;pr.per_page=1;pr.show=proteins#tc:rf) | 16 | 63784 | 2 (1) | 0.05 | McKusick-Kaufman/Bardet-Biedl syndromes putative chaperonin OS=Homo sapiens OX=9606 GN=MKKS PE=1 SV=1 | |
| [Q9NP60](http://sale-depta-pc/mascot/cgi/master_results_2.pl?file=20181219%2FF002798.dat;pr.eh=118%2C118p;pr.page=118;pr.per_page=1;pr.show=proteins#tc:rf) | 16 | 79702 | 1 (1) | 0.04 | X-linked interleukin-1 receptor accessory protein-like 2 OS=Homo sapiens OX=9606 GN=IL1RAPL2 PE=2 SV=1 | |
| [Q96Q42](http://sale-depta-pc/mascot/cgi/master_results_2.pl?file=20181219%2FF002798.dat;pr.eh=119%2C119p;pr.page=119;pr.per_page=1;pr.show=proteins#tc:rf) | 16 | 185342 | 1 (1) | 0.02 | Alsin OS=Homo sapiens OX=9606 GN=ALS2 PE=1 SV=2 | |
| [Q8NC42](http://sale-depta-pc/mascot/cgi/master_results_2.pl?file=20181219%2FF002796.dat;pr.eh=35%2C35p;pr.page=35;pr.per_page=1;pr.show=proteins#tc:rf) | 15 | 43707 | 2 (2) | 0.08 | E3 ubiquitin-protein ligase RNF149 OS=Homo sapiens OX=9606 GN=RNF149 PE=2 SV=2 | |
| [Q6ZXV5](http://sale-depta-pc/mascot/cgi/master_results_2.pl?file=20181219%2FF002798.dat;pr.eh=120%2C120p;pr.page=120;pr.per_page=1;pr.show=proteins#tc:rf) | 13 | 104854 | 5 (1) | 0.03 | Transmembrane and TPR repeat-containing protein 3 OS=Homo sapiens OX=9606 GN=TMTC3 PE=1 SV | |
