# Supplementary material for: The circular RNA circ-GRB10 participates in the molecular circuitry inhibiting human intervertebral disc degeneration
Source: Cell Death Dis. 2020 Aug 13;11(8):612. doi: 10.1038/s41419-020-02882-3 (PMC7426430; doi:10.1038/s41419-020-02882-3)
Supplement: Supplementary file 6 — Supplementary Table S2 [file 41419_2020_2882_MOESM6_ESM.docx]

Supplementary Table S2. Enrichment analysis demonstrated that these 143 protein

| **Biological pathway** | **No. of genes in the dataset** | **Fold enrichment** | **P-value (Hypergeometric test)** | **genes mapped (from input data set)** |
| --- | --- | --- | --- | --- |
| Gene Expression | 32 | 6.05203875 | 1.05475E-17 | PRPF4; HNRNPM; HNRNPH1; HNRNPH2; HNRNPF; FUS; HNRNPA1; ALYREF; YBX1; HNRNPU; SNRPD3; RAE1; RBMX; EIF1AX; SNW1; RPS25; HNRNPA2B1; PCBP2; RPS14; RPS6; RPL11; HNRNPK; RPL22; NUP153; RPL24; HNRNPD; NUDT21; RPS27A; RPL12; SRSF3; HNRNPA0; NOTCH2; |
| Processing of Capped Intron-Containing Pre-mRNA | 21 | 10.88014821 | 9.05761E-17 | PRPF4; HNRNPM; HNRNPH1; HNRNPH2; HNRNPF; FUS; HNRNPA1; ALYREF; YBX1; HNRNPU; SNRPD3; RAE1; RBMX; HNRNPA2B1; PCBP2; HNRNPK; NUP153; HNRNPD; NUDT21; SRSF3; HNRNPA0; |
| mRNA Processing | 21 | 9.563526238 | 1.36685E-15 | PRPF4; HNRNPM; HNRNPH1; HNRNPH2; HNRNPF; FUS; HNRNPA1; ALYREF; YBX1; HNRNPU; SNRPD3; RAE1; RBMX; HNRNPA2B1; PCBP2; HNRNPK; NUP153; HNRNPD; NUDT21; SRSF3; HNRNPA0; |
| Formation and Maturation of mRNA Transcript | 21 | 8.116151855 | 3.99004E-14 | PRPF4; HNRNPM; HNRNPH1; HNRNPH2; HNRNPF; FUS; HNRNPA1; ALYREF; YBX1; HNRNPU; SNRPD3; RAE1; RBMX; HNRNPA2B1; PCBP2; HNRNPK; NUP153; HNRNPD; NUDT21; SRSF3; HNRNPA0; |
| mRNA Splicing - Major Pathway | 19 | 12.69629684 | 1.59934E-16 | PRPF4; HNRNPM; HNRNPH1; HNRNPH2; HNRNPF; FUS; HNRNPA1; ALYREF; YBX1; HNRNPU; SNRPD3; RBMX; HNRNPA2B1; PCBP2; HNRNPK; HNRNPD; NUDT21; SRSF3; HNRNPA0; |
| mRNA Splicing | 19 | 12.69629684 | 1.59934E-16 | PRPF4; HNRNPM; HNRNPH1; HNRNPH2; HNRNPF; FUS; HNRNPA1; ALYREF; YBX1; HNRNPU; SNRPD3; RBMX; HNRNPA2B1; PCBP2; HNRNPK; HNRNPD; NUDT21; SRSF3; HNRNPA0; |
| Metabolism of RNA | 17 | 4.326152789 | 2.41272E-07 | SMG8; HSPA8; HSPA1A; HSPB1; SNRPD3; RAE1; RPS25; RPS14; RPS6; RPL11; RPL22; NUP153; RPL24; HNRNPD; YWHAZ; RPS27A; RPL12; |
| Regulation of CDC42 activity | 16 | 1.489854208 | 0.065011623 | MTOR; HSP90AA1; HSP90B1; HSPA8; HNRNPA1; ENO1; HSPB1; PRDX1; ARHGEF2; PKM; NUP153; YWHAZ; RBBP4; ALDOA; TAB2; OBSCN; |
| CDC42 signaling events | 16 | 1.515506987 | 0.057306666 | MTOR; HSP90AA1; HSP90B1; HSPA8; HNRNPA1; ENO1; HSPB1; PRDX1; ARHGEF2; PKM; NUP153; YWHAZ; RBBP4; ALDOA; TAB2; OBSCN; |
| Metabolism of proteins | 14 | 3.865813678 | 1.18421E-05 | TUBB2B; TUBB4B; ACTB; EIF1AX; RPS25; RPS14; RPS6; RPL11; RPL22; RPL24; RPS27A; CALR; RPL12; ALG13; |
| Metabolism of mRNA | 14 | 4.27881031 | 3.6477E-06 | SMG8; HSPA8; HSPA1A; HSPB1; RPS25; RPS14; RPS6; RPL11; RPL22; RPL24; HNRNPD; YWHAZ; RPS27A; RPL12; |
| Influenza Life Cycle | 13 | 6.887009375 | 3.49417E-08 | HSP90AA1; HSPA1A; RAE1; RPS25; RPS14; RPS6; RPL11; RPL22; NUP153; RPL24; RPS27A; CALR; RPL12; |
| Influenza Infection | 13 | 6.641062323 | 5.41662E-08 | HSP90AA1; HSPA1A; RAE1; RPS25; RPS14; RPS6; RPL11; RPL22; NUP153; RPL24; RPS27A; CALR; RPL12; |
| Integrin-linked kinase signaling | 13 | 1.421713943 | 0.121722825 | HSP90AA1; HSP90B1; HSPA8; HNRNPA1; ENO1; HSPB1; PRDX1; PKM; NUP153; YWHAZ; RBBP4; ALDOA; TAB2; |
| AP-1 transcription factor network | 13 | 1.497262743 | 0.089988337 | HSP90AA1; HSP90B1; HSPA8; HNRNPA1; ENO1; HSPB1; PRDX1; PKM; NUP153; YWHAZ; RBBP4; ALDOA; TAB2; |
| Diabetes pathways | 12 | 3.973639512 | 4.06149E-05 | HSP90B1; HSPA5; RPS25; RPS14; RPS6; RPL11; IGF2BP2; RPL22; RPL24; RPS27A; CALR; RPL12; |
| Developmental Biology | 11 | 1.821431462 | 0.037499424 | HSP90AB1; HSP90AA1; RPS25; RPS14; RPS6; RPL11; CAP1; RPL22; RPL24; RPS27A; RPL12; |
| L13a-mediated translational silencing of Ceruloplasmin expression | 9 | 6.312499523 | 1.07388E-05 | EIF1AX; RPS25; RPS14; RPS6; RPL11; RPL22; RPL24; RPS27A; RPL12; |
| 3' -UTR-mediated translational regulation | 9 | 6.312499523 | 1.07388E-05 | EIF1AX; RPS25; RPS14; RPS6; RPL11; RPL22; RPL24; RPS27A; RPL12; |
| GTP hydrolysis and joining of the 60S ribosomal subunit | 9 | 6.25121907 | 1.16336E-05 | EIF1AX; RPS25; RPS14; RPS6; RPL11; RPL22; RPL24; RPS27A; RPL12; |
| Translation | 9 | 5.503273877 | 3.26352E-05 | EIF1AX; RPS25; RPS14; RPS6; RPL11; RPL22; RPL24; RPS27A; RPL12; |
| Eukaryotic Translation Initiation | 9 | 5.85345038 | 1.98727E-05 | EIF1AX; RPS25; RPS14; RPS6; RPL11; RPL22; RPL24; RPS27A; RPL12; |
| Cap-dependent Translation Initiation | 9 | 5.85345038 | 1.98727E-05 | EIF1AX; RPS25; RPS14; RPS6; RPL11; RPL22; RPL24; RPS27A; RPL12; |
| Formation of a pool of free 40S subunits | 9 | 6.998566203 | 4.56516E-06 | EIF1AX; RPS25; RPS14; RPS6; RPL11; RPL22; RPL24; RPS27A; RPL12; |
| Influenza Viral RNA Transcription and Replication | 9 | 6.570126276 | 7.72256E-06 | HSP90AA1; RPS25; RPS14; RPS6; RPL11; RPL22; RPL24; RPS27A; RPL12; |
| Nonsense-Mediated Decay | 9 | 6.191116973 | 1.25913E-05 | SMG8; RPS25; RPS14; RPS6; RPL11; RPL22; RPL24; RPS27A; RPL12; |
| Nonsense Mediated Decay Enhanced by the Exon Junction Complex | 9 | 6.191116973 | 1.25913E-05 | SMG8; RPS25; RPS14; RPS6; RPL11; RPL22; RPL24; RPS27A; RPL12; |
| Regulation of beta-cell development | 8 | 5.300146389 | 0.000119881 | RPS25; RPS14; RPS6; RPL11; RPL22; RPL24; RPS27A; RPL12; |
| Regulation of gene expression in beta cells | 8 | 5.840922472 | 6.00411E-05 | RPS25; RPS14; RPS6; RPL11; RPL22; RPL24; RPS27A; RPL12; |
| Insulin Synthesis and Processing | 8 | 4.336556409 | 0.000477463 | RPS25; RPS14; RPS6; RPL11; RPL22; RPL24; RPS27A; RPL12; |
| Eukaryotic Translation Termination | 8 | 6.980475692 | 1.63119E-05 | RPS25; RPS14; RPS6; RPL11; RPL22; RPL24; RPS27A; RPL12; |
| Eukaryotic Translation Elongation | 8 | 6.734134943 | 2.12862E-05 | RPS25; RPS14; RPS6; RPL11; RPL22; RPL24; RPS27A; RPL12; |
| Peptide chain elongation | 8 | 6.980475692 | 1.63119E-05 | RPS25; RPS14; RPS6; RPL11; RPL22; RPL24; RPS27A; RPL12; |
| Viral mRNA Translation | 8 | 6.980475692 | 1.63119E-05 | RPS25; RPS14; RPS6; RPL11; RPL22; RPL24; RPS27A; RPL12; |
| Nonsense Mediated Decay Independent of the Exon Junction Complex | 8 | 6.579345035 | 2.52648E-05 | RPS25; RPS14; RPS6; RPL11; RPL22; RPL24; RPS27A; RPL12; |
| Cell Cycle, Mitotic | 7 | 1.580390356 | 0.154132515 | PCM1; TUBB; TUBB4B; HSP90AA1; NUMA1; RPS27A; RBBP4; |
| Immune System | 7 | 0.95975086 | 0.603729482 | MTOR; HSP90AB1; PCBP2; YWHAZ; RPS27A; CALR; TAB2; |
| Signal Transduction | 6 | 0.355568482 | 0.999744451 | MTOR; SNW1; PLCB4; RPS6; RPS27A; NOTCH2; |
| Regulation of mRNA Stability by Proteins that Bind AU-rich Elements | 6 | 4.129701777 | 0.003202897 | HSPA8; HSPA1A; HSPB1; HNRNPD; YWHAZ; RPS27A; |
| Regulation of cytoplasmic and nuclear SMAD2/3 signaling | 6 | 1.408249834 | 0.253864326 | HSPA8; HSPB1; NUP153; YWHAZ; RBBP4; TAB2; |
| TGF-beta receptor signaling | 6 | 1.408249834 | 0.253864326 | HSPA8; HSPB1; NUP153; YWHAZ; RBBP4; TAB2; |
| ALK1 pathway | 6 | 1.325669831 | 0.299866675 | HSPA8; HSPB1; NUP153; YWHAZ; RBBP4; TAB2; |
| ATM pathway | 6 | 1.399075867 | 0.258610707 | HSPA1A; DDX5; RPL11; YWHAZ; RPS27L; RBBP4; |
| Regulation of nuclear SMAD2/3 signaling | 6 | 1.408249834 | 0.253864326 | HSPA8; HSPB1; NUP153; YWHAZ; RBBP4; TAB2; |
| ALK1 signaling events | 6 | 1.338058882 | 0.292480891 | HSPA8; HSPB1; NUP153; YWHAZ; RBBP4; TAB2; |
| TNF alpha/NF-kB | 6 | 2.511726108 | 0.032025887 | HSP90AB1; HSP90AA1; HSPB1; YWHAZ; POLR1D; TAB2; |
| Mitotic M-M/G1 phases | 5 | 1.479529841 | 0.250009963 | PCM1; TUBB; TUBB4B; HSP90AA1; RPS27A; |
| DNA Replication | 5 | 1.37182873 | 0.301429048 | PCM1; TUBB; TUBB4B; HSP90AA1; RPS27A; |
| Mitotic G2-G2/M phases | 5 | 3.616412655 | 0.012211872 | PCM1; TUBB; TUBB4B; HSP90AA1; NUMA1; |
| G2/M Transition | 5 | 3.729413779 | 0.010781133 | PCM1; TUBB; TUBB4B; HSP90AA1; NUMA1; |
| Recruitment of mitotic centrosome proteins and complexes | 5 | 5.114426752 | 0.002832496 | PCM1; TUBB; TUBB4B; HSP90AA1; NUMA1; |
| Centrosome maturation | 5 | 5.114426752 | 0.002832496 | PCM1; TUBB; TUBB4B; HSP90AA1; NUMA1; |
| Adaptive Immune System | 5 | 1.510742234 | 0.236883622 | MTOR; YWHAZ; RPS27A; CALR; TAB2; |
| Ribosomal scanning and start codon recognition | 5 | 6.629531882 | 0.000881644 | EIF1AX; RPS25; RPS14; RPS6; RPS27A; |
| Translation initiation complex formation | 5 | 6.629531882 | 0.000881644 | EIF1AX; RPS25; RPS14; RPS6; RPS27A; |
| Activation of the mRNA upon binding of the cap-binding complex and eIFs, and subsequent binding to 43S | 5 | 6.50901685 | 0.000959243 | EIF1AX; RPS25; RPS14; RPS6; RPS27A; |
| Formation of the ternary complex, and subsequently, the 43S complex | 5 | 7.616698935 | 0.000461746 | EIF1AX; RPS25; RPS14; RPS6; RPS27A; |
| Destabilization of mRNA by AUF1 (hnRNP D0) | 5 | 5.042402717 | 0.003014008 | HSPA8; HSPA1A; HSPB1; HNRNPD; RPS27A; |
| Transcription | 5 | 2.011465743 | 0.103199612 | ALYREF; SNRPD3; NUDT21; SRSF3; POLR1D; |
| C-MYC pathway | 5 | 2.402932803 | 0.057048155 | HSP90AA1; ENO1; HSPD1; NCL; RPL11; |
| Validated targets of C-MYC transcriptional activation | 5 | 4.419960708 | 0.005318824 | HSP90AA1; ENO1; HSPD1; NCL; RPL11; |
| ATR signaling pathway | 5 | 1.43218678 | 0.271395101 | HSPA1A; DDX5; RPL11; YWHAZ; RPS27L; |
| Platelet activation, signaling and aggregation | 4 | 2.076601348 | 0.127167195 | HSPA5; PFN1; CAP1; YWHAZ; |
| Hemostasis | 4 | 0.764224293 | 0.778568021 | HSPA5; PFN1; CAP1; YWHAZ; |
| Mitotic Prophase | 4 | 4.621702178 | 0.010768995 | PCM1; TUBB; TUBB4B; HSP90AA1; |
| Golgi Cisternae Pericentriolar Stack Reorganization | 4 | 4.621702178 | 0.010768995 | PCM1; TUBB; TUBB4B; HSP90AA1; |
| M Phase | 4 | 1.813757054 | 0.179562347 | PCM1; TUBB; TUBB4B; HSP90AA1; |
| Post-Elongation Processing of the Transcript | 4 | 6.663374845 | 0.002912064 | ALYREF; SNRPD3; NUDT21; SRSF3; |
| Host Interactions of HIV factors | 4 | 2.032421474 | 0.13456307 | RAE1; SLC25A5; NUP153; RPS27A; |
| HIV Infection | 4 | 1.411712487 | 0.31606529 | RAE1; SLC25A5; NUP153; RPS27A; |
| Loss of Nlp from mitotic centrosomes | 4 | 4.697455369 | 0.010180849 | PCM1; TUBB; TUBB4B; HSP90AA1; |
| Loss of proteins required for interphase microtubule organizationÃÂ from the centrosome | 4 | 4.697455369 | 0.010180849 | PCM1; TUBB; TUBB4B; HSP90AA1; |
| Transport of Mature mRNA derived from an Intron-Containing Transcript | 4 | 5.618344483 | 0.005417846 | ALYREF; RAE1; NUP153; SRSF3; |
| Transport of Mature Transcript to Cytoplasm | 4 | 5.20981189 | 0.007088143 | ALYREF; RAE1; NUP153; SRSF3; |
| RNA Polymerase II Transcription | 4 | 2.837261183 | 0.052459732 | ALYREF; SNRPD3; NUDT21; SRSF3; |
| Cleavage of Growing Transcript in the Termination Region | 4 | 6.663374845 | 0.002912064 | ALYREF; SNRPD3; NUDT21; SRSF3; |
| RNA Polymerase II Transcription Termination | 4 | 6.663374845 | 0.002912064 | ALYREF; SNRPD3; NUDT21; SRSF3; |
| Metabolism of carbohydrates | 4 | 3.114789176 | 0.039412467 | RAE1; PC; NUP153; TKT; |
| Innate Immune System | 4 | 1.565989575 | 0.25321794 | HSP90AB1; PCBP2; RPS27A; TAB2; |
| Wnt signaling network | 4 | 1.432887116 | 0.306509042 | HNRNPA1; PRDX1; YWHAZ; TAB2; |
| Signaling events mediated by HDAC Class I | 4 | 2.535985772 | 0.073095023 | TUBA1B; HSP90AA1; NUP153; RBBP4; |
| Glypican 3 network | 4 | 1.391154566 | 0.32565005 | HNRNPA1; PRDX1; YWHAZ; TAB2; |
| Regulation of Telomerase | 4 | 4.213964889 | 0.014760313 | MTOR; HSP90AA1; NCL; RBBP4; |
| Noncanonical Wnt signaling pathway | 4 | 1.57459344 | 0.250144841 | HNRNPA1; PRDX1; YWHAZ; TAB2; |
| Hypoxic and oxygen homeostasis regulation of HIF-1-alpha | 4 | 3.581949157 | 0.025279734 | HSP90AA1; ENO1; PKM; ALDOA; |
| TNF receptor signaling pathway | 4 | 0.958468787 | 0.608364287 | HSPB1; NUMA1; YWHAZ; TAB2; |
| p53 pathway | 4 | 1.51627825 | 0.271820427 | HSPA1A; DDX5; RPL11; RPS27L; |
| Syndecan-4-mediated signaling events | 4 | 1.371186795 | 0.33525691 | HNRNPA1; PRDX1; YWHAZ; TAB2; |
| Axon guidance | 3 | 0.982249611 | 0.596729289 | HSP90AB1; HSP90AA1; CAP1; |
| Platelet degranulation | 3 | 7.680203042 | 0.006732965 | HSPA5; PFN1; CAP1; |
| Response to elevated platelet cytosolic Ca2+ | 3 | 5.514547224 | 0.016824087 | HSPA5; PFN1; CAP1; |
| Post-Elongation Processing of Intron-Containing pre-mRNA | 3 | 6.325271603 | 0.011580416 | ALYREF; NUDT21; SRSF3; |
| mRNA 3'-end processing | 3 | 6.325271603 | 0.011580416 | ALYREF; NUDT21; SRSF3; |
| Interactions of Vpr with host cellular proteins | 3 | 6.325271603 | 0.011580416 | RAE1; SLC25A5; NUP153; |
| Transport of Mature mRNA Derived from an Intronless Transcript | 3 | 6.144601177 | 0.012539978 | ALYREF; RAE1; NUP153; |
| Transport of the SLBP independent Mature mRNA | 3 | 6.720477576 | 0.009792946 | ALYREF; RAE1; NUP153; |
| Transport of the SLBP Dependant Mature mRNA | 3 | 6.516888434 | 0.010664859 | ALYREF; RAE1; NUP153; |
| Transport of Mature mRNAs Derived from Intronless Transcripts | 3 | 5.97396521 | 0.013543865 | ALYREF; RAE1; NUP153; |
| Unfolded Protein Response | 3 | 3.414100733 | 0.057646428 | HSP90B1; HSPA5; CALR; |
| Activation of Chaperones by ATF6-alpha | 3 | 23.87596972 | 0.000209177 | HSP90B1; HSPA5; CALR; |
| Protein folding | 3 | 7.680203042 | 0.006732965 | TUBB2B; TUBB4B; ACTB; |
| Export of Viral Ribonucleoproteins from Nucleus | 3 | 6.937197266 | 0.008964272 | HSPA1A; RAE1; NUP153; |
| Membrane Trafficking | 3 | 2.560677148 | 0.112724337 | HSPA8; CLINT1; RPS27A; |
| Metabolism of amino acids and derivatives | 3 | 1.144207687 | 0.492581412 | MCCC1; MCCC2; RPS27A; |
| Metabolism of non-coding RNA | 3 | 4.301589426 | 0.032344153 | SNRPD3; RAE1; NUP153; |
| snRNP Assembly | 3 | 4.301589426 | 0.032344153 | SNRPD3; RAE1; NUP153; |
| Transmembrane transport of small molecules | 3 | 0.569092054 | 0.906252209 | ALB; RAE1; NUP153; |
| SLC-mediated transmembrane transport | 3 | 0.870905984 | 0.678276027 | ALB; RAE1; NUP153; |
| Metabolism of lipids and lipoproteins | 3 | 0.837019911 | 0.704334985 | ALB; ACACA; ABCB4; |
| Nucleotide-binding domain, leucine rich repeat containing receptor (NLR) signaling pathways | 3 | 5.001685357 | 0.021830988 | HSP90AB1; RPS27A; TAB2; |
| Signaling events mediated by HDAC Class II | 3 | 5.659628709 | 0.015685697 | TUBA1B; HSP90AA1; NUP153; |
| Stabilization and expansion of the E-cadherin adherens junction | 3 | 0.782235145 | 0.747203953 | HNRNPA1; PRDX1; YWHAZ; |
| HIF-1-alpha transcription factor network | 3 | 3.258937846 | 0.064501522 | ENO1; PKM; ALDOA; |
| N-cadherin signaling events | 3 | 0.857027558 | 0.688893781 | HNRNPA1; PRDX1; YWHAZ; |
| IL1-mediated signaling events | 3 | 0.919287583 | 0.641977819 | HSPB1; YWHAZ; TAB2; |
| Posttranslational regulation of adherens junction stability and dissassembly | 3 | 0.931225866 | 0.633213982 | HNRNPA1; PRDX1; YWHAZ; |
| Canonical Wnt signaling pathway | 3 | 1.387797479 | 0.369662887 | HNRNPA1; PRDX1; YWHAZ; |
| E-cadherin signaling in the nascent adherens junction | 3 | 0.782235145 | 0.747203953 | HNRNPA1; PRDX1; YWHAZ; |
| Regulation of nuclear beta catenin signaling and target gene transcription | 3 | 1.593381877 | 0.292634142 | HNRNPA1; PRDX1; YWHAZ; |
| p38 MAPK signaling pathway | 3 | 1.138153998 | 0.496142073 | HSPB1; YWHAZ; TAB2; |
| IL2-mediated signaling events | 3 | 1.870467674 | 0.217237723 | MTOR; HSP90AA1; RPS6; |
| E-cadherin signaling events | 3 | 0.768267159 | 0.75820989 | HNRNPA1; PRDX1; YWHAZ; |
| Androgen-mediated signaling | 3 | 1.654661082 | 0.273495122 | HSP90AA1; HNRNPA1; PRDX1; |
| Direct p53 effectors | 3 | 1.55874565 | 0.304168075 | HSPA1A; DDX5; RPS27L; |
| IL2 signaling events mediated by PI3K | 3 | 3.210304241 | 0.066866966 | MTOR; HSP90AA1; RPS6; |
| Regulation of Androgen receptor activity | 3 | 1.991690466 | 0.19192157 | HSP90AA1; HNRNPA1; PRDX1; |
| BMP receptor signaling | 3 | 0.951827296 | 0.618288079 | HSPB1; YWHAZ; TAB2; |
| TGFBR | 3 | 1.72084223 | 0.254509811 | HSPA8; SNW1; NUP153; |
| Nuclear import of Rev protein | 2 | 4.78684513 | 0.065383424 | RAE1; NUP153; |
| Interactions of Rev with host cellular proteins | 2 | 4.35180922 | 0.077276926 | RAE1; NUP153; |
| Semaphorin interactions | 2 | 2.244230938 | 0.225326583 | HSP90AB1; HSP90AA1; |
| Sema3A PAK dependent Axon repulsion | 2 | 9.570501157 | 0.018053832 | HSP90AB1; HSP90AA1; |
| Processing of Capped Intronless Pre-mRNA | 2 | 6.243077895 | 0.040479188 | SNRPD3; NUDT21; |
| Post-Elongation Processing of Intronless pre-mRNA | 2 | 6.243077895 | 0.040479188 | SNRPD3; NUDT21; |
| Neuronal System | 2 | 0.764072243 | 0.745222734 | KCNG2; HSPA8; |
| Vpr-mediated nuclear import of PICs | 2 | 4.632480566 | 0.0692744 | RAE1; NUP153; |
| Rev-mediated nuclear export of HIV-1 RNA | 2 | 4.487760774 | 0.073239831 | RAE1; NUP153; |
| Late Phase of HIV Life Cycle | 2 | 1.561278365 | 0.370042329 | RAE1; NUP153; |
| HIV Life Cycle | 2 | 1.394556085 | 0.424512804 | RAE1; NUP153; |
| Signalling by NGF | 2 | 1.004497744 | 0.599165132 | MTOR; RPS27A; |
| mTOR signalling | 2 | 5.318519895 | 0.054186071 | MTOR; RPS6; |
| S6K1-mediated signalling | 2 | 13.04752247 | 0.009805721 | MTOR; RPS6; |
| mTORC1-mediated signalling | 2 | 13.04752247 | 0.009805721 | MTOR; RPS6; |
| Signaling by FGFR | 2 | 1.511980027 | 0.385111132 | MTOR; RPS27A; |
| IRS-mediated signalling | 2 | 2.816177659 | 0.159367119 | MTOR; RPS6; |
| PI3K Cascade | 2 | 3.77935339 | 0.098444002 | MTOR; RPS6; |
| PKB-mediated events | 2 | 5.12864057 | 0.05783631 | MTOR; RPS6; |
| IRS-related events | 2 | 2.709926851 | 0.169283554 | MTOR; RPS6; |
| Signaling by Insulin receptor | 2 | 1.818165072 | 0.303367068 | MTOR; RPS6; |
| Insulin receptor signalling cascade | 2 | 2.564778117 | 0.184347189 | MTOR; RPS6; |
| Signaling by EGFR | 2 | 1.465699647 | 0.400027218 | MTOR; RPS27A; |
| Mitotic G1-G1/S phases | 2 | 1.026021158 | 0.587514706 | RPS27A; RBBP4; |
| Signaling by Notch | 2 | 6.837373744 | 0.03420755 | SNW1; NOTCH2; |
| NICD traffics to nucleus | 2 | 13.04752247 | 0.009805721 | SNW1; NOTCH2; |
| Generic Transcription Pathway | 2 | 4.103205437 | 0.085555259 | SNW1; NOTCH2; |
| Notch-HLH transcription pathway | 2 | 13.04752247 | 0.009805721 | SNW1; NOTCH2; |
| Asparagine N-linked glycosylation | 2 | 1.865384007 | 0.292973256 | CALR; ALG13; |
| Post-chaperonin tubulin folding pathway | 2 | 11.96113425 | 0.011660583 | TUBB2B; TUBB4B; |
| Post-translational protein modification | 2 | 1.260005459 | 0.476513301 | CALR; ALG13; |
| NEP/NS2 Interacts with the Cellular Export Machinery | 2 | 4.951851857 | 0.061569755 | RAE1; NUP153; |
| Transport of Ribonucleoproteins into the Host Nucleus | 2 | 4.951851857 | 0.061569755 | RAE1; NUP153; |
| trans-Golgi Network Vesicle Budding | 2 | 4.35180922 | 0.077276926 | HSPA8; CLINT1; |
| Clathrin derived vesicle budding | 2 | 4.35180922 | 0.077276926 | HSPA8; CLINT1; |
| Golgi Associated Vesicle Biogenesis | 2 | 5.318519895 | 0.054186071 | HSPA8; CLINT1; |
| Branched-chain amino acid catabolism | 2 | 8.445221773 | 0.022965509 | MCCC1; MCCC2; |
| Glucose transport | 2 | 3.77935339 | 0.098444002 | RAE1; NUP153; |
| Regulation of Glucokinase by Glucokinase Regulatory Protein | 2 | 4.951851857 | 0.061569755 | RAE1; NUP153; |
| Hexose transport | 2 | 3.590432951 | 0.107321584 | RAE1; NUP153; |
| Fatty acid, triacylglycerol, and ketone body metabolism | 2 | 1.730553215 | 0.324073176 | ACACA; ABCB4; |
| Class I MHC mediated antigen processing & presentation | 2 | 1.422168324 | 0.414777409 | RPS27A; CALR; |
| Apoptosis | 2 | 0.909140069 | 0.653756185 | RPS27A; DSG1; |
| TRIF mediated TLR3 signaling | 2 | 2.564778117 | 0.184347189 | RPS27A; TAB2; |
| Toll Like Receptor 3 (TLR3) Cascade | 2 | 2.564778117 | 0.184347189 | RPS27A; TAB2; |
| TRAF6 Mediated Induction of proinflammatory cytokines | 2 | 2.872489949 | 0.154451822 | RPS27A; TAB2; |
| Toll Like Receptor 5 (TLR5) Cascade | 2 | 2.393821402 | 0.204717325 | RPS27A; TAB2; |
| Toll Like Receptor 7/8 (TLR7/8) Cascade | 2 | 2.31661381 | 0.214997659 | RPS27A; TAB2; |
| MyD88 dependent cascade initiated on endosome | 2 | 2.393821402 | 0.204717325 | RPS27A; TAB2; |
| TRAF6 mediated induction of NFkB and MAP kinases upon TLR7/8 or 9 activation | 2 | 2.434387771 | 0.199598809 | RPS27A; TAB2; |
| Toll Like Receptor 9 (TLR9) Cascade | 2 | 2.209709619 | 0.230505967 | RPS27A; TAB2; |
| IRAK2 mediated activation of TAK1 complex upon TLR7/8 or 9 stimulation | 2 | 14.35097126 | 0.008096067 | RPS27A; TAB2; |
| Activated TLR4 signalling | 2 | 1.994906574 | 0.266926218 | RPS27A; TAB2; |
| MyD88:Mal cascade initiated on plasma membrane | 2 | 2.31661381 | 0.214997659 | RPS27A; TAB2; |
| Toll Like Receptor 4 (TLR4) Cascade | 2 | 1.889925304 | 0.287769146 | RPS27A; TAB2; |
| NFkB and MAP kinases activation mediated by TLR4 signaling repertoire | 2 | 2.659752312 | 0.174281235 | RPS27A; TAB2; |
| MyD88-independent cascade initiated on plasma membrane | 2 | 2.476352739 | 0.194496627 | RPS27A; TAB2; |
| Toll Receptor Cascades | 2 | 1.595969585 | 0.359918761 | RPS27A; TAB2; |
| Antigen processing-Cross presentation | 2 | 1.670192098 | 0.339506443 | RPS27A; CALR; |
| ER-Phagosome pathway | 2 | 1.795440849 | 0.308555027 | RPS27A; CALR; |
| Toll Like Receptor 10 (TLR10) Cascade | 2 | 2.393821402 | 0.204717325 | RPS27A; TAB2; |
| IRAK2 mediated activation of TAK1 complex | 2 | 14.35097126 | 0.008096067 | RPS27A; TAB2; |
| MyD88 cascade initiated on plasma membrane | 2 | 2.434387771 | 0.199598809 | RPS27A; TAB2; |
| RIG-I/MDA5 mediated induction of IFN-alpha/beta pathways | 2 | 2.143757982 | 0.240888271 | PCBP2; RPS27A; |
| Cytokine Signaling in Immune system | 2 | 0.744278651 | 0.758308043 | YWHAZ; TAB2; |
| Negative regulators of RIG-I/MDA5 signaling | 2 | 5.12864057 | 0.05783631 | PCBP2; RPS27A; |
| Signaling by Interleukins | 2 | 1.561278365 | 0.370042329 | YWHAZ; TAB2; |
| TRAF6 mediated induction of TAK1 complex | 2 | 10.25362044 | 0.015788807 | RPS27A; TAB2; |
| Toll Like Receptor 2 (TLR2) Cascade | 2 | 2.209709619 | 0.230505967 | RPS27A; TAB2; |
| Toll Like Receptor TLR1:TLR2 Cascade | 2 | 2.244230938 | 0.225326583 | RPS27A; TAB2; |
| Toll Like Receptor TLR6:TLR2 Cascade | 2 | 2.244230938 | 0.225326583 | RPS27A; TAB2; |
| NOD1/2 Signaling Pathway | 2 | 5.522999706 | 0.050622088 | RPS27A; TAB2; |
| sucrose degradation | 2 | 20.49261375 | 0.003882757 | ALDOC; ALDOA; |
| Notch-mediated HES/HEY network | 2 | 1.52806321 | 0.380104476 | ENO1; NOTCH2; |
| p38 signaling mediated by MAPKAP kinases | 2 | 6.837373744 | 0.03420755 | HSPB1; YWHAZ; |
| Coregulation of Androgen receptor activity | 2 | 2.354584861 | 0.209850741 | HNRNPA1; PRDX1; |
| Signaling mediated by p38-alpha and p38-beta | 2 | 2.872489949 | 0.154451822 | HSPB1; YWHAZ; |
| Regulation of RAC1 activity | 2 | 0.700713245 | 0.787393184 | ARHGEF2; OBSCN; |
| Regulation of p38-alpha and p38-beta | 2 | 0.875880875 | 0.673900895 | HSPB1; YWHAZ; |
| C-MYB transcription factor network | 2 | 1.709953843 | 0.329228026 | HSPA8; TAB2; |
| RAC1 signaling pathway | 2 | 0.700713245 | 0.787393184 | ARHGEF2; OBSCN; |
| Notch signaling pathway | 2 | 1.52806321 | 0.380104476 | ENO1; NOTCH2; |
| RhoA signaling pathway | 2 | 0.700713245 | 0.787393184 | ARHGEF2; OBSCN; |
| Regulation of RhoA activity | 2 | 0.700713245 | 0.787393184 | ARHGEF2; OBSCN; |
| Alpha6Beta4Integrin | 2 | 2.992152101 | 0.144716036 | MTOR; YWHAZ; |
| NOTCH | 2 | 2.519789903 | 0.189412251 | SNW1; NOTCH2; |
| Vif-mediated degradation of APOBEC3G | 1 | 1.045992719 | 0.62374545 | RPS27A; |
| Vpu mediated degradation of CD4 | 1 | 1.077211722 | 0.612873208 | RPS27A; |
| Signaling by Robo receptor | 1 | 3.137068992 | 0.277201534 | CAP1; |
| Role of Abl in Robo-Slit signaling | 1 | 8.011538014 | 0.119164626 | CAP1; |
| GP1b-IX-V activation signalling | 1 | 8.011538014 | 0.119164626 | YWHAZ; |
| APC-Cdc20 mediated degradation of Nek2A | 1 | 3.137068992 | 0.277201534 | RPS27A; |
| Processing of Intronless Pre-mRNAs | 1 | 5.152316738 | 0.179182985 | NUDT21; |
| SLBP independent Processing of Histone Pre-mRNAs | 1 | 7.211184566 | 0.131505577 | SNRPD3; |
| SLBP Dependent Processing of Replication-Dependent Histone Pre-mRNAs | 1 | 6.556217757 | 0.143675562 | SNRPD3; |
| Striated Muscle Contraction | 1 | 2.40533014 | 0.345354803 | DMD; |
| Muscle contraction | 1 | 1.443390472 | 0.506997258 | DMD; |
| Transmission across Chemical Synapses | 1 | 0.596512334 | 0.821182597 | HSPA8; |
| Neurotransmitter Release Cycle | 1 | 2.775238658 | 0.3072289 | HSPA8; |
| GABA synthesis, release, reuptake and degradation | 1 | 4.243618901 | 0.213234137 | HSPA8; |
| Voltage gated Potassium channels | 1 | 1.850396245 | 0.423718602 | KCNG2; |
| Potassium Channels | 1 | 0.949663959 | 0.659474175 | KCNG2; |
| Vpr-mediated induction of apoptosis by mitochondrial outer membrane permeabilization | 1 | 23.98138123 | 0.041393412 | SLC25A5; |
| PI3K/AKT activation | 1 | 2.40533014 | 0.345354803 | MTOR; |
| p75 NTR receptor-mediated signalling | 1 | 1.850396245 | 0.423718602 | RPS27A; |
| Cell death signalling via NRAGE, NRIF and NADE | 1 | 3.435695264 | 0.256472114 | RPS27A; |
| NRIF signals cell death from the nucleus | 1 | 5.152316738 | 0.179182985 | RPS27A; |
| Release of eIF4E | 1 | 12.01064185 | 0.08109208 | MTOR; |
| S6K1 signalling | 1 | 14.40797555 | 0.068043202 | RPS6; |
| NF-kB is activated and signals survival | 1 | 5.548344159 | 0.167511972 | RPS27A; |
| p75NTR recruits signalling complexes | 1 | 5.548344159 | 0.167511972 | RPS27A; |
| p75NTR signals via NF-kB | 1 | 4.508679419 | 0.202041865 | RPS27A; |
| NGF signalling via TRKA from the plasma membrane | 1 | 0.674553383 | 0.781395189 | MTOR; |
| PIP3 activates AKT signaling | 1 | 3.435695264 | 0.256472114 | MTOR; |
| EGFR downregulation | 1 | 2.672490096 | 0.316961316 | RPS27A; |
| GAB1 signalosome | 1 | 2.255043971 | 0.363632246 | MTOR; |
| Spry regulation of FGF signaling | 1 | 4.508679419 | 0.202041865 | RPS27A; |
| PI-3K cascade | 1 | 2.672490096 | 0.316961316 | MTOR; |
| Negative regulation of FGFR signaling | 1 | 4.243618901 | 0.213234137 | RPS27A; |
| Downstream signaling of activated FGFR | 1 | 1.093530639 | 0.607321194 | MTOR; |
| Assembly of the pre-replicative complex | 1 | 0.869581466 | 0.691845042 | RPS27A; |
| DNA Replication Pre-Initiation | 1 | 0.736495842 | 0.751318227 | RPS27A; |
| M/G1 Transition | 1 | 0.736495842 | 0.751318227 | RPS27A; |
| Autodegradation of Cdh1 by Cdh1:APC/C | 1 | 1.202865481 | 0.572317126 | RPS27A; |
| APC/C:Cdc20 mediated degradation of Cyclin B | 1 | 3.279598251 | 0.266909258 | RPS27A; |
| APC/C:Cdc20 mediated degradation of Securin | 1 | 1.093530639 | 0.607321194 | RPS27A; |
| APC/C:Cdh1 mediated degradation of Cdc20 and other APC/C:Cdh1 targeted proteins in late mitosis/early G1 | 1 | 1.045992719 | 0.62374545 | RPS27A; |
| Regulation of mitotic cell cycle | 1 | 0.891049963 | 0.68292009 | RPS27A; |
| Recruitment of NuMA to mitotic centrosomes | 1 | 6.010321191 | 0.155676924 | NUMA1; |
| APC/C-mediated degradation of cell cycle proteins | 1 | 0.891049963 | 0.68292009 | RPS27A; |
| Regulation of APC/C activators between G1/S and early anaphase | 1 | 0.925316722 | 0.669051409 | RPS27A; |
| APC/C:Cdc20 mediated degradation of mitotic proteins | 1 | 1.016532284 | 0.634315725 | RPS27A; |
| Activation of APC/C and APC/C:Cdc20 mediated degradation of mitotic proteins | 1 | 1.002415741 | 0.639490225 | RPS27A; |
| SCF-beta-TrCP mediated degradation of Emi1 | 1 | 1.0310521 | 0.62906781 | RPS27A; |
| Ubiquitin-dependent degradation of Cyclin D | 1 | 1.093530639 | 0.607321194 | RPS27A; |
| Ubiquitin-dependent degradation of Cyclin D1 | 1 | 1.093530639 | 0.607321194 | RPS27A; |
| SCF(Skp2)-mediated degradation of p27/p21 | 1 | 1.077211722 | 0.612873208 | RPS27A; |
| Cyclin E associated events during G1/S transition | 1 | 0.925316722 | 0.669051409 | RPS27A; |
| G1/S Transition | 1 | 0.616904175 | 0.810609292 | RPS27A; |
| S Phase | 1 | 0.596512334 | 0.821182597 | RPS27A; |
| Cyclin A:Cdk2-associated events at S phase entry | 1 | 0.801954866 | 0.72117034 | RPS27A; |
| G1 Phase | 1 | 2.327763867 | 0.354557483 | RPS27A; |
| G0 and Early G1 | 1 | 3.435695264 | 0.256472114 | RBBP4; |
| Cyclin D associated events in G1 | 1 | 2.327763867 | 0.354557483 | RPS27A; |
| mRNA Splicing - Minor Pathway | 1 | 1.718256546 | 0.4477196 | SNRPD3; |
| Binding of RNA by Insulin-like Growth Factor-2 mRNA Binding Proteins (IGF2BPs/IMPs/VICKZs) | 1 | 23.98138123 | 0.041393412 | IGF2BP2; |
| Activation of Chaperones by IRE1alpha | 1 | 1.60373156 | 0.470731893 | HSPA5; |
| PERK regulated gene expression | 1 | 6.556217757 | 0.143675562 | HSPA5; |
| Nucleosome assembly | 1 | 2.061809697 | 0.390106492 | RBBP4; |
| Deposition of New CENPA-containing Nucleosomes at the Centromere | 1 | 2.061809697 | 0.390106492 | RBBP4; |
| Removal of licensing factors from origins | 1 | 0.849123133 | 0.700521555 | RPS27A; |
| Chromosome Maintenance | 1 | 1.045992719 | 0.62374545 | RBBP4; |
| Regulation of DNA replication | 1 | 0.820179042 | 0.713085411 | RPS27A; |
| Association of licensing factors with the pre-replicative complex | 1 | 4.809057795 | 0.19069221 | RPS27A; |
| CDT1 association with the CDC6:ORC:origin complex | 1 | 0.975327084 | 0.649623028 | RPS27A; |
| Synthesis of DNA | 1 | 0.662177392 | 0.787574021 | RPS27A; |
| Switching of origins to a post-replicative state | 1 | 0.869581466 | 0.691845042 | RPS27A; |
| Orc1 removal from chromatin | 1 | 0.869581466 | 0.691845042 | RPS27A; |
| CDK-mediated phosphorylation and removal of Cdc6 | 1 | 1.093530639 | 0.607321194 | RPS27A; |
| Mature Notch receptor traffics to plasma membrane | 1 | 18.00098691 | 0.054811159 | NOTCH2; |
| Notch receptor binds with a ligand | 1 | 9.011730026 | 0.106650336 | NOTCH2; |
| Transport of Notch receptor precursor to golgi | 1 | 18.00098691 | 0.054811159 | NOTCH2; |
| Maturation of Notch precursor via proteolytic cleavage | 1 | 14.40797555 | 0.068043202 | NOTCH2; |
| Signaling by GPCR | 1 | 0.090228819 | 0.999994216 | PLCB4; |
| Receptor-ligand binding initiates the second proteolytic cleavage of Notch receptor | 1 | 8.011538014 | 0.119164626 | NOTCH2; |
| A third proteolytic cleavage releases NICD | 1 | 9.011730026 | 0.106650336 | NOTCH2; |
| Signaling by PDGF | 1 | 0.925316722 | 0.669051409 | MTOR; |
| Downstream signal transduction | 1 | 0.962324457 | 0.65458332 | MTOR; |
| Signaling by SCF-KIT | 1 | 1.093530639 | 0.607321194 | MTOR; |
| GPCR downstream signaling | 1 | 0.136193577 | 0.999591339 | PLCB4; |
| G alpha (q) signalling events | 1 | 0.601482856 | 0.818595302 | PLCB4; |
| Opioid Signalling | 1 | 1.361704537 | 0.52756555 | PLCB4; |
| PLC beta mediated events | 1 | 1.850396245 | 0.423718602 | PLCB4; |
| G-protein mediated events | 1 | 1.804147901 | 0.431831346 | PLCB4; |
| TCR signaling | 1 | 1.53550218 | 0.4855441 | TAB2; |
| Signaling by Wnt | 1 | 0.962324457 | 0.65458332 | RPS27A; |
| Degradation of beta-catenin by the destruction complex | 1 | 0.962324457 | 0.65458332 | RPS27A; |
| The citric acid (TCA) cycle and respiratory electron transport | 1 | 0.611676616 | 0.813309176 | COX6C; |
| DNA Repair | 1 | 0.680916494 | 0.778239477 | RPS27A; |
| Bile acid and bile salt metabolism | 1 | 2.672490096 | 0.316961316 | ALB; |
| Recycling of bile acids and salts | 1 | 6.556217757 | 0.143675562 | ALB; |
| Regulation of Lipid Metabolism by Peroxisome proliferator-activated receptor alpha (PPARalpha) | 1 | 2.255043971 | 0.363632246 | ABCB4; |
| Tetrahydrobiopterin (BH4) synthesis, recycling, salvage and regulation | 1 | 6.010321191 | 0.155676924 | HSP90AA1; |
| eNOS activation and regulation | 1 | 4.007993198 | 0.224271184 | HSP90AA1; |
| Metabolism of nitric oxide | 1 | 4.007993198 | 0.224271184 | HSP90AA1; |
| eNOS activation | 1 | 8.011538014 | 0.119164626 | HSP90AA1; |
| Regulation of the Fanconi anemia pathway | 1 | 9.011730026 | 0.106650336 | RPS27A; |
| Fanconi Anemia pathway | 1 | 3.279598251 | 0.266909258 | RPS27A; |
| Stabilization of p53 | 1 | 1.045992719 | 0.62374545 | RPS27A; |
| p53-Dependent G1 DNA Damage Response | 1 | 1.002415741 | 0.639490225 | RPS27A; |
| p53-Dependent G1/S DNA damage checkpoint | 1 | 1.002415741 | 0.639490225 | RPS27A; |
| G1/S DNA Damage Checkpoints | 1 | 0.949663959 | 0.659474175 | RPS27A; |
| Cell Cycle Checkpoints | 1 | 0.611676616 | 0.813309176 | RPS27A; |
| Autodegradation of the E3 ubiquitin ligase COP1 | 1 | 1.061372703 | 0.618347602 | RPS27A; |
| p53-Independent G1/S DNA damage checkpoint | 1 | 1.0310521 | 0.62906781 | RPS27A; |
| Ubiquitin Mediated Degradation of Phosphorylated Cdc25A | 1 | 1.045992719 | 0.62374545 | RPS27A; |
| p53-Independent DNA Damage Response | 1 | 1.0310521 | 0.62906781 | RPS27A; |
| Biosynthesis of the N-glycan precursor (dolichol lipid-linked oligosaccharide, LLO) and transfer to a nascent protein | 1 | 2.40533014 | 0.345354803 | ALG13; |
| N-glycan trimming in the ER and Calnexin/Calreticulin cycle | 1 | 5.548344159 | 0.167511972 | CALR; |
| Calnexin/calreticulin cycle | 1 | 6.010321191 | 0.155676924 | CALR; |
| Folding of actin by CCT/TriC | 1 | 8.011538014 | 0.119164626 | ACTB; |
| Cooperation of Prefoldin and TriC/CCT in actin and tubulin folding | 1 | 4.809057795 | 0.19069221 | ACTB; |
| Chaperonin-mediated protein folding | 1 | 4.508679419 | 0.202041865 | ACTB; |
| Assembly of Viral Components at the Budding Site | 1 | 35.91241667 | 0.027787383 | CALR; |
| Viral RNP Complexes in the Host Cell Nucleus | 1 | 35.91241667 | 0.027787383 | HSPA1A; |
| Virus Assembly and Release | 1 | 35.91241667 | 0.027787383 | CALR; |
| vRNP Assembly | 1 | 35.91241667 | 0.027787383 | HSP90AA1; |
| Meiotic Synapsis | 1 | 1.53550218 | 0.4855441 | HSPA2; |
| Meiosis | 1 | 1.002415741 | 0.639490225 | HSPA2; |
| Endosomal Sorting Complex Required For Transport (ESCRT) | 1 | 2.577078097 | 0.326558539 | RPS27A; |
| Lysosome Vesicle Biogenesis | 1 | 4.809057795 | 0.19069221 | HSPA8; |
| Integration of energy metabolism | 1 | 0.869581466 | 0.691845042 | TKT; |
| Regulation of ornithine decarboxylase (ODC) | 1 | 1.0310521 | 0.62906781 | RPS27A; |
| Insulin effects increased synthesis of Xylulose-5-Phosphate | 1 | 35.91241667 | 0.027787383 | TKT; |
| Destabilization of mRNA by KSRP | 1 | 3.797157154 | 0.235155134 | YWHAZ; |
| Respiratory electron transport | 1 | 0.949663959 | 0.659474175 | COX6C; |
| Respiratory electron transport, ATP synthesis by chemiosmotic coupling, and heat production by uncoupling proteins. | 1 | 0.793143144 | 0.725127923 | COX6C; |
| Glucose metabolism | 1 | 2.00455311 | 0.398686928 | PC; |
| Circadian Clock | 1 | 2.255043971 | 0.363632246 | RPS27A; |
| Fatty Acyl-CoA Biosynthesis | 1 | 4.007993198 | 0.224271184 | ACACA; |
| Triglyceride Biosynthesis | 1 | 2.577078097 | 0.326558539 | ACACA; |
| Lipoprotein metabolism | 1 | 2.672490096 | 0.316961316 | ALB; |
| HDL-mediated lipid transport | 1 | 4.809057795 | 0.19069221 | ALB; |
| Gluconeogenesis | 1 | 3.607394178 | 0.245888087 | PC; |
| Pentose phosphate pathway (hexose monophosphate shunt) | 1 | 12.01064185 | 0.08109208 | TKT; |
| Cell-cell junction organization | 1 | 1.60373156 | 0.470731893 | PARD6G; |
| Cell-Cell communication | 1 | 0.616904175 | 0.810609292 | PARD6G; |
| Cell junction organization | 1 | 1.045992719 | 0.62374545 | PARD6G; |
| Tight junction interactions | 1 | 4.007993198 | 0.224271184 | PARD6G; |
| Lipid digestion, mobilization, and transport | 1 | 1.568875408 | 0.478189954 | ALB; |
| Transport of vitamins, nucleosides, and related molecules | 1 | 2.40533014 | 0.345354803 | ALB; |
| Transport of organic anions | 1 | 7.211184566 | 0.131505577 | ALB; |
| Antigen processing: Ubiquitination & Proteasome degradation | 1 | 1.045992719 | 0.62374545 | RPS27A; |
| CD28 co-stimulation | 1 | 2.00455311 | 0.398686928 | MTOR; |
| CD28 dependent PI3K/Akt signaling | 1 | 4.243618901 | 0.213234137 | MTOR; |
| Downstream TCR signaling | 1 | 2.255043971 | 0.363632246 | TAB2; |
| Costimulation by the CD28 family | 1 | 1.387886128 | 0.520805512 | MTOR; |
| RNA Polymerase I, RNA Polymerase III, and Mitochondrial Transcription | 1 | 0.78452296 | 0.729029962 | POLR1D; |
| RNA Polymerase I Transcription | 1 | 1.288769104 | 0.547285072 | POLR1D; |
| RNA Polymerase I Promoter Clearance | 1 | 1.336492455 | 0.534231292 | POLR1D; |
| Regulation of Apoptosis | 1 | 0.962324457 | 0.65458332 | RPS27A; |
| Regulation of activated PAK-2p34 by proteasome mediated degradation | 1 | 1.093530639 | 0.607321194 | RPS27A; |
| RNA Polymerase I Promoter Escape | 1 | 3.607394178 | 0.245888087 | POLR1D; |
| RNA Polymerase I Transcription Initiation | 1 | 3.006412224 | 0.28735093 | POLR1D; |
| RNA Polymerase I Chain Elongation | 1 | 1.503519215 | 0.492795761 | POLR1D; |
| RNA Polymerase I Transcription Termination | 1 | 3.435695264 | 0.256472114 | POLR1D; |
| Cdc20:Phospho-APC/C mediated degradation of Cyclin A | 1 | 1.045992719 | 0.62374545 | RPS27A; |
| Apoptotic cleavage of cellular proteins | 1 | 1.850396245 | 0.423718602 | DSG1; |
| Apoptotic cleavage of cell adhesion proteins | 1 | 6.010321191 | 0.155676924 | DSG1; |
| Apoptotic execution phase | 1 | 1.503519215 | 0.492795761 | DSG1; |
| Cross-presentation of soluble exogenous antigens (endosomes) | 1 | 1.077211722 | 0.612873208 | RPS27A; |
| Rap1 signalling | 1 | 7.211184566 | 0.131505577 | YWHAZ; |
| Antigen Presentation: Folding, assembly and peptide loading of class I MHC | 1 | 3.137068992 | 0.277201534 | CALR; |
| Interleukin-1 signaling | 1 | 1.850396245 | 0.423718602 | TAB2; |
| Interleukin-3, 5 and GM-CSF signaling | 1 | 2.488243968 | 0.336022424 | YWHAZ; |
| The NLRP3 inflammasome | 1 | 6.556217757 | 0.143675562 | HSP90AB1; |
| Inflammasomes | 1 | 4.243618901 | 0.213234137 | HSP90AB1; |
| superpathway of serine and glycine biosynthesis I | 1 | 18.00098691 | 0.054811159 | PHGDH; |
| serine biosynthesis | 1 | 35.91241667 | 0.027787383 | PHGDH; |
| dolichyl-diphosphooligosaccharide biosynthesis | 1 | 10.29728352 | 0.0939603 | ALG13; |
| biotin-carboxyl carrier protein assembly | 1 | 23.98138123 | 0.041393412 | ACACA; |
| leucine degradation I | 1 | 10.29728352 | 0.0939603 | MCCC2; |
| pyruvate fermentation to lactate | 1 | 14.40797555 | 0.068043202 | LDHB; |
| pentose phosphate pathway (non-oxidative branch) | 1 | 12.01064185 | 0.08109208 | TKT; |
| pentose phosphate pathway | 1 | 7.211184566 | 0.131505577 | TKT; |
| CXCR3-mediated signaling events | 1 | 1.950390638 | 0.407148001 | MTOR; |
| ErbB2/ErbB3 signaling events | 1 | 1.899078072 | 0.415491353 | MTOR; |
| IL6-mediated signaling events | 1 | 1.53550218 | 0.4855441 | HSP90B1; |
| Signaling events mediated by the Hedgehog family | 1 | 1.1103516 | 0.601690473 | RBBP4; |
| Integrins in angiogenesis | 1 | 1.127698133 | 0.595979942 | HSP90AA1; |
| Sumoylation by RanBP2 regulates transcriptional repression | 1 | 4.809057795 | 0.19069221 | NUP153; |
| FOXA transcription factor networks | 1 | 0.891049963 | 0.68292009 | ALB; |
| Neurotrophic factor-mediated Trk receptor signaling | 1 | 0.714621894 | 0.761772632 | YWHAZ; |
| Aurora B signaling | 1 | 1.760155023 | 0.43983116 | NCL; |
| Caspase cascade in apoptosis | 1 | 1.387886128 | 0.520805512 | NUMA1; |
| FOXA2 and FOXA3 transcription factor networks | 1 | 1.678306382 | 0.455498197 | ALB; |
| Signaling events mediated by TCPTP | 1 | 0.78452296 | 0.729029962 | MTOR; |
| Reelin signaling pathway | 1 | 2.488243968 | 0.336022424 | ARHGEF2; |
| Signaling by Aurora kinases | 1 | 0.736495842 | 0.751318227 | NCL; |
| Alpha4 beta1 integrin signaling events | 1 | 2.186730006 | 0.37258085 | YWHAZ; |
| VEGFR1 specific signals | 1 | 2.488243968 | 0.336022424 | HSP90AA1; |
| p75(NTR)-mediated signaling | 1 | 0.40550507 | 0.92145708 | YWHAZ; |
| FoxO family signaling | 1 | 1.47284141 | 0.499946349 | YWHAZ; |
| FAS (CD95) signaling pathway | 1 | 0.555218502 | 0.842885897 | NUMA1; |
| Signaling events mediated by HDAC Class III | 1 | 1.850396245 | 0.423718602 | TUBA1B; |
| FOXM1 transcription factor network | 1 | 1.718256546 | 0.4477196 | HSPA1A; |
| Glucocorticoid receptor signaling | 1 | 0.849123133 | 0.700521555 | HSP90AA1; |
| Hedgehog signaling events mediated by Gli proteins | 1 | 1.503519215 | 0.492795761 | RBBP4; |
| Insulin-mediated glucose transport | 1 | 2.488243968 | 0.336022424 | YWHAZ; |
| Glucocorticoid receptor regulatory network | 1 | 0.902186695 | 0.67836226 | HSP90AA1; |
| ErbB4 signaling events | 1 | 2.255043971 | 0.363632246 | TAB2; |
| Role of Calcineurin-dependent NFAT signaling in lymphocytes | 1 | 0.759751158 | 0.740410478 | YWHAZ; |
| IL4-mediated signaling events | 1 | 1.164069626 | 0.584314963 | MTOR; |
| Signaling events mediated by PTP1B | 1 | 1.387886128 | 0.520805512 | YBX1; |
| CXCR4-mediated signaling events | 1 | 0.379895571 | 0.93401308 | MTOR; |
| a6b1 and a6b4 Integrin signaling | 1 | 2.061809697 | 0.390106492 | YWHAZ; |
| Regulation of retinoblastoma protein | 1 | 1.093530639 | 0.607321194 | RBBP4; |
| E2F transcription factor network | 1 | 0.988685899 | 0.644592326 | RBBP4; |
| IL12-mediated signaling events | 1 | 0.650247343 | 0.793580139 | MTOR; |
| Endogenous TLR signaling | 1 | 1.266163086 | 0.553675658 | HSPD1; |
| Signaling events mediated by PRL | 1 | 3.137068992 | 0.277201534 | TUBA1B; |
| Trk receptor signaling mediated by PI3K and PLC-gamma | 1 | 1.145595263 | 0.590188483 | YWHAZ; |
| AndrogenReceptor | 1 | 0.925316722 | 0.669051409 | CALR; |
