# Supplementary material for: The circular RNA circ-GRB10 participates in the molecular circuitry inhibiting human intervertebral disc degeneration
Source: Cell Death Dis. 2020 Aug 13;11(8):612. doi: 10.1038/s41419-020-02882-3 (PMC7426430; doi:10.1038/s41419-020-02882-3)
Supplement: Supplementary file 7 — Supplementary Table S3 [file 41419_2020_2882_MOESM7_ESM.docx]

Supplementary Tables S3. 19 predicted miRNAs regulate FUS

| Database Names | Total | miRNAs |
| --- | --- | --- |
| Microt4, PITA, RNAhybrid, Targetscan, miRanda | 19 | hsa-miR-320c |
|  |  | hsa-miR-141-3p |
|  |  | hsa-miR-200a-3p |
|  |  | hsa-miR-200b-3p |
|  |  | hsa-miR-320d |
|  |  | hsa-miR-7-5p |
|  |  | hsa-miR-579-3p |
|  |  | hsa-miR-1197 |
|  |  | hsa-miR-513c-5p |
|  |  | hsa-let-7g-3p |
|  |  | hsa-miR-200c-3p |
|  |  | hsa-miR-548d-3p |
|  |  | hsa-miR-429 |
|  |  | hsa-miR-522-3p |
|  |  | hsa-miR-548p |
|  |  | hsa-miR-1262 |
|  |  | hsa-miR-320a |
|  |  | hsa-miR-1225-5p |
|  |  | hsa-miR-320b |
