# Supplementary material for: The circular RNA circ-GRB10 participates in the molecular circuitry inhibiting human intervertebral disc degeneration
Source: Cell Death Dis. 2020 Aug 13;11(8):612. doi: 10.1038/s41419-020-02882-3 (PMC7426430; doi:10.1038/s41419-020-02882-3)
Supplement: Supplementary file 8 — Supplementary Table S4 [file 41419_2020_2882_MOESM8_ESM.docx]

Supplementary Tables S4. Primers used for quantitative real-time PCR

| Name |  | Sequence |
| --- | --- | --- |
| Circ-GRB10 | Forward | 5′-GCCGCCGCAAAGCAGATATTC-3′ |
|  | Reverse | 5′- ACAGACTCCAGCAGGGTCAG-3′ |
| ERBB2 | Forward | 5′-TGTGACTGCCTGTCCCTACAA-3′ |
|  | Reverse | 5′-CCAGACCATAGCACACTCGG-3′ |
| FUS | Forward | ATGGCCTCAAACGATTATACCCA |
|  | Reverse | GTAACTCTGCTGTCCGTAGGG |
| Collagen-II | Forward | TCCCAGAACATCACCTACCAC |
|  | Reverse | CCATCCTTCAGGGCAGTGTA |
| Aggrecan | Forward | ACAATGCCCAAGACTACCAG |
|  | Reverse | GTGCCAGATCATCACCACA |
| MMP-13 | Forward | GGAAGACCTCCAGTTTGCAGAGC |
|  | Reverse | GCTGCATTCTCCTTCAGGATTCC |
| ADAMT-5 | Forward | AGTTTGCCTATCGTCACTGTAAT |
|  | Reverse | GTAGATGGCCCTCTTCCCT |
| miR-141-3p | Forward | CATCCGATTAACACTGTCTGGTAA |
|  | Reverse | TATGGTTGTTCTGCTCTCTGTCTC |
| GAPDH | Forward | GCACCGTCAAGGCTGAGAAC |
|  | Reverse | GGATCTCGCTCCTGGAAGATG |
| U6 | Forward | CTCGCTTCGGCAGCACA |
|  | Reverse | AACGCTTCACGAATTTGCGT |
